# DTARNU-Net: Dense Tiered Attention Residual Nested U-Net for CT Liver Tumor Segmentation

**DOI:** 10.3390/bioengineering13090992

**Published:** 2026-08-27

**Authors:** Kumar P, Robert P, Parthasarathy Ramadass, Mohd Anul Haq

**Affiliations:** 1 Department of Computer Science and Engineering, Rajalakshmi Engineering College, Chennai 602105, India; 2 Department of Computing Technologies, College of Engineering and Technology, SRM Institute of Science and Technology, Chennai 603203, India; 3 Department of Computer Science and Engineering, Vel Tech Rangarajan Dr. Sagunthala R&D Institute of Science and Technology, Chennai 600055, India; 4 Department of Computer Science, College of Computer and Information Sciences, Majmaah University, Al Majmaah 11952, Saudi Arabia

**Keywords:** liver tumor segmentation, computer tomography, improved U-Net, optimization algorithm, semantic segmentation, medical image processing

## Abstract

Liver tumor segmentation is a significant task in clinical imaging that involves detecting liver tumors and distinguishing them from the surrounding liver tissue in CT scans. Precision segmentation performs important roles in the initial detection of liver cancer, treatment planning, and monitoring disease development, which also supports doctors, facilitating surgeries and radiation therapy more efficiently. Meanwhile, clinical imaging and segmentation algorithms have been enhanced over the years. The currently prevailing state-of-the-art methods still face multiple difficulties, though, in obtaining precision and reliability in their outcomes. Tumors with irregular shapes, variable sizes, and densities similar to those of surrounding tissues often lead to segmentation inaccuracies and potential misdiagnoses. In this work, we tackle these challenges by developing an advanced process for precise liver tumor segmentation by utilizing CT images from the LiTS dataset. The proposed DTARNU-Net was developed, trained, validated, and evaluated exclusively using the Liver Tumor Segmentation (LiTS) benchmark dataset. No experiments were conducted on the 3D-IRCADbI dataset in this study. All quantitative and qualitative results presented in the manuscript correspond to the LiTS dataset. The LiTS dataset contains contrast-enhanced abdominal CT scans with expert-annotated liver and tumor masks. The proposed model was evaluated using patient-level training, validation, and testing partitions (9:2:2 ratio), and all experiments were independently repeated five times. Statistical significance was assessed using paired Student’s t-test (*p* < 0.05), and the results confirmed that the performance improvements over competing methods are statistically significant. We introduce a novel three-level pre-processing approach that significantly enhances image quality through histogram equalization, noise removal, smoothing, and sharpening. Our approach is embodied in the Dense Tiered Attention Residual Nested U-Net (DTARNU-Net), a sophisticated model combining the strengths of a Siamese network and a nested U-Net architecture. This model incorporates the ACON-ReLU residual convolution block (A-R), which improves recognition accuracy in regions with subtle changes, reducing missed detection. The presented method enhances trait collaboration and spatial data by utilizing the Brownian Motion-based Butterfly Optimization Algorithm (BM-BOA). This algorithm efficiently integrates low-level trait details with high-level semantic data. The Dense Tiered Attention Residual Module (DTSRM) additionally improves these traits to obtain more precise segmentation. The model achieved segmentation robustness of 96.78% for liver segmentation and 97.00% for liver tumor segmentation on the LiTS dataset. These outcomes indicate that the presented method performs better than the prevailing state-of-the-art methods and has the capability to help computer-assisted detection and treatment by furnishing more precise and reliable liver tumor segmentation.

## 1. Introduction

Globally, liver cancer is one of the most important types of cancer and is associated with high mortality rates. Initial detection and precise diagnosis are necessary for enhancing patient survival, making liver tumor segmentation a significant task in clinical imaging [1,2]. Liver tumor segmentation detects and marks tumors in clinical images, allowing specialists to establish the tumor size, shape, and location. This data helps treatment planning, disease supervision, and medical decision-making. Common imaging modalities utilized for liver tumor segmentation involve Computed Tomography (CT), Magnetic Resonance Imaging (MRI), and Ultrasound (US), each providing particular advantages in examining liver tumors [3,4]. CT is the most broadly utilized imaging method for liver tumor diagnosis, due to its high-resolution cross-sectional images, and clearly differentiates the tumor from healthy liver tissue based on variations in tissue density [5,6]. The utilization of contrast-enhanced CT further enhances tumor visibility, assuring more precise segmentation [7]. MRI offers superior soft-tissue contrast and functional data, like diffusion and perfusion features, which support the detailed assessment of liver tumors. Moreover, MRI is more costly, takes longer to perform, and is not available in all clinical settings [8]. Ultrasound is a low-cost, non-invasive, and real-time imaging method generally utilized for liver evaluations. While its lower image resolution and dependence on operator experience may decrease segmentation precision, it remains valuable for liver cancer screening and for supporting biopsy and ablation processes, particularly when integrated with CT or MRI [9,10].

CT imaging offers high-resolution images that explicitly represent the liver anatomy, which is useful in cases involving small tumors and those placed near challenging anatomical structures. This precision is significant for detection, treatment planning, and surgical decision-making. Contrast-enhanced CT, moreover, enhances the visibility of tumors by highlighting the variations between healthy liver tissue and cancerous tissue, leading to more accurate tumor identification and segmentation [11,12]. CT scanners are broadly available in hospitals, and their fast imaging process makes them perfect for routine clinical practice. In addition, CT creates three-dimensional (3D) images, facilitating the tasks of medical professionals in precisely examining the sizes, shapes, and locations of tumors, which is particularly supportive in cases of liver surgery and transplantation. Importantly, machine learning (ML) and deep learning (DL) have enhanced the automation and precision of liver tumor segmentation utilizing CT, MRI, and Ultrasound images. The prevailing ML methods, like Support Vector Machine (SVM), Random Forest (RF), and K-Nearest Neighbors (KNN), depend on individually separated traits, utilizing texture, shape, and intensity to detect tumors [13,14,15]. Moreover, the techniques frequently struggle with differences in tumor visualization resulting from variations in imaging techniques, patient anatomy, and tumor traits.

Deep learning, specifically, the involvement of Convolutional Neural Networks (CNNs), has also benefited liver tumor segmentation by automatically learning meaningful traits directly from clinical images. Popular models like U-Net, V-Net, and Fully Convolutional Networks (FCNs) have obtained superior segmentation performance. These models need large numbers of precision-annotated medical images, and are costly and time-consuming to support. Their proficiency can also be disturbed by image noise, artifacts, and variations in imaging protocols, resulting in unstable segmentation among clinical datasets [16,17]. However, deep learning models need substantial computational resources and frequently require modification when used with other imaging modalities like MRI and Ultrasound. Regardless of the difficulties, CT-based liver tumor segmentation remains a robust and efficient approach due to its high image quality, superior contrast-based improvements, three-dimensional visualization capacity, and broad use in medical practice. Meanwhile, CT imaging remains a highly effective modality for liver tumor segmentation because of its high spatial resolution, contrast enhancement capabilities, and widespread availability; the integration of ML and DL approaches has further improved segmentation accuracy and efficiency. Despite the modern developments in liver tumor segmentation, multiple difficulties remain. The proficiency of the prevailing methods relies heavily on the accessibility of large annotated datasets and is disturbed by differences in image quality, image protocols, and computational requirements. Moreover, multiple deep learning models de-emphasize comprehensibility, providing more challenges for their medical application [18,19,20]. Thus, more research is required to promote segmentation models that are more accurate, reliable, understandable, and able to normalize across various datasets and imaging modalities.

Multiple attention-based U-Net models, including Attention U-Net, CBAM-UNet, ResUNet, ERes-UNet++, and ARU-Net, have enhanced liver tumor segmentation. Moreover, most of these models utilize only a single attention mechanism or a fixed trait fusion strategy. As a result, they frequently face difficulties in obtaining multi-scale traits, maintaining tumor boundaries, and precisely segmenting small or abnormal liver tumors. Further, the currently prevailing methods normally join traits directly without refining the extra data, which can provide redundant traits and decrease segmentation precision.

To address these thresholds, the DTARNU-Net presented here proposes two important components. The first is the Dense Tiered Attention Residual Module (DTARM), which optimizes hierarchical traits, utilizing residual learning together with channel and spatial attention. The second is the Brownian Motion-based Butterfly Optimization Algorithm (BM-BOA), which dynamically maximizes multi-level features before they are fused. By integrating these two modules, DTARNU-Net enhances multi-scale feature learning, retains tumor thresholds more efficiently, and reaches higher liver tumor segmentation precision than the currently prevailing attention-based U-Net models.

**A.**  **Research Contribution.** The important advancements of the presented work are summarized as follows:
  - Three-level Pre-Processing Approach: A novel three-level pre-processing architecture is presented, aiming to enhance the quality of CT images. The method combines histogram equalization, noise removal, image smoothing, and sharpening to improve tumor clarity and retain significant anatomical details, leading to better liver tumor segmentation.
  - Novel Dense Tiered Attention Residual Nested U-Net (DTARNU-Net): A new DTARUN-Net framework is presented for liver tumor segmentation. As opposed to conventional Attention U-Net or UNet++ frameworks that support a single attention pathway, the explored network integrates Dense Tiered Attention Residual Modules (DTARM) inside a nested encoder–decoder structure. This structure gradually optimizes multi-scale traits while conserving correct anatomical boundaries, resulting in more precise tumor segmentation.
  - Novel Brownian Motion-Based Butterfly Optimization (BM-BOA): Existing attention-based U-Net models directly fuse encoder and decoder features without adaptive optimization. The proposed BM-BOA performs optimization of multi-level feature representations before feature fusion. This calibration enhances trait learning, decreases redundant data, and improves the identification of irregular liver tumor thresholds.
  - Novel Dense Tiered Attention Residual Module (DTARM): An innovative DTARM is proposed for the enhancement of trait learning. As opposed to conventional CBAM-based or SE-based attention modules that perform the attention function only once, and to the whole traits map, DTARM separates trait channels into hierarchical groups and fine-tunes them, utilizing the remaining learning jointly with channel and spatial attention. The refined traits are then integrated through dense links, maintaining fine threshold information at the time of computational effectiveness.
  - Scientific Novelty: The important innovation of the presented DTARNU-Net depends on the integrated utilization of BM-BOA and DTARM. BM-BOA first maintains hierarchical encoder traits before trait fusion, while DTARM gradually refines these streamlining traits through dense residual attention learning, in contrast to the prevailing attention-based U-Net models that employ attention-only trait maximization, trait calibration, and multi-scale trait accumulation. This collaborative strategy enhances threshold localization, retains small tumor structures, enhances segmentation reliability, and protects computational effectiveness.

As a result, DTARNU-Net is more than a synthesis of the currently prevailing methods. The significant optimization is the hierarchical, streamlined architecture, where BM-BOA and DTARM work in a combined manner to improve multi-scale traits learning and attention-guided traits optimization. This integrated design supports more precise tumor threshold delineation and better detection of small liver lesions than the currently prevailing U-Net-based segmentation methods.

**B.**  **Paper Organization**
  The remainder of this paper is arranged as follows. Section 2 provides an in-depth review of related work and indicates significant difficulties in the field. Section 3 presents the DTARNU-Net model, furnishing theoretical, mathematical, and visual designs. Section 4 introduces the experimental outcomes, which involve the dataset, integrated information, workout examination, numerical calibration, and graphical presentation. Section 5 contains discussions. Finally, Section 6 concludes the research and indicates potential directions for future research.

## 2. Related Works

Manjunath R V et al. [21] mostly focused on DL approaches for liver segmentation and associated tumors, beginning with abdominal CT scan images to reduce the time and number of determinations essential for the identification of liver disease. Here, the algorithm is based on the altered architecture of ResUNet. The research presents an automatic technique based on semantic segmentation, employing CNN and liver visualizations from CT images, with the hepatic portion divided into segments. Rahman H et al. [22] addressed this problem by recommending a hybrid ResUNet model which integrates the ResNet and UNet models for better performance. In this study, the two overlapping models were primarily implemented for liver segmentation and ROI calculation. The liver is segmented by employing an abdominal CT image to train the model. The public 3D dataset IRCADB01 is used to measure the presented approach; this dataset is based on CT volume slices of individuals with liver tumors. Özcan F et al. [23] presented the AIM-Unet approach. It is a hybridization of the CNN-based Unet and Inception approaches. Four distinct CT liver image datasets, three of which were pre-existing and one of which was created precisely for this learning, were implemented in experimental investigations utilizing CHAOS, LIST, and 3DIRCADb. The accuracy (ACC), JSC, DSC, and segmentation results designated by a professional were associated with the results attained by employing the presented approach. Hussain M et al. [24] emphasized machine learning (ML) techniques for multi-class liver tumor classification. Four tumor classes were encompassed in the dataset. Later, the images were transformed into grayscale, and then histogram equalization was employed to boost the contrast. The Gabor filter was implemented to diminish noise, and an image sharpening technique was employed to augment image quality. Out of 55 features, 20 optimal features were nominated for classification by implementing the CFS technique. Li J et al. [25] employed UNet++ structure as the baseline of his study. An effective attention module is presented which utilizes the spatial information and depth of the feature map to improve the influence of irregular trial circulation. In the last step, Dice loss is implemented to optimize network parameters by substituting the cross-entropy loss function. Lei T et al. [26] presented DefED-Net for the purpose of segmenting the liver and liver tumors. The presented approach utilizes two complementary features. It utilizes multiscale dilation rate within the Ladder-ASPP unit approach to absorb improved background data. Zhang Y et al. [27] considered a spatial aggregation module (SAM) to fully utilize cross-phase information, which encourages per-pixel interactions across various phases. Moreover, the paper proposed an Uncertain Region Inpainting Module (URIM) which controls nearby discriminating characteristics to refine uncertain pixels. Sabir M W et al. [28] implemented the 3D-IRCADb01 dataset to generate a highly dense ResU-Net network architecture on CT scans. The residual block and U-Net architecture excerpt more information from the input data than the conventional U-Net network, a crucial aspect of ResU-Net. Image pre-processing techniques are employed before the images are guided into the deep neural network; these include data augmentation, the Hounsfield windowing unit, and histogram equalization. Han K et al. [29] proposed adding a pseudo-labelling procedure under an extremely low-annotation data paradigm to the existing DSS method for segmentation of liver CT images. To be more detailed, the output features of the pre-trained network based on labeled images were synthesized by matching. Subsequently, pseudo labels for the unlabeled images were formed by measuring the separations between every class representation and the unlabeled feature vectors. The research also utilizes a number of methods to optimize pseudo labels to increase their quality. Aghamohammadi A et al. [30] offered a straightforward yet effective procedure based on a cascading CNN for segmentation of tumor and liver. Initially, the Z-Score procedure is employed to normalize the input image. Further details regarding the liver and tumor boundaries then originate from the normalized image. Additionally, a unique method called LDOG is suggested for determining some important aspects within the image. The presented image encoding is quite good at recognizing the border of the liver, even in the areas which are touching proximate organs. The original image and two additional acquired images are then normalized.

Li L et al. [31] presented the RDCTrans U-Net for the segmentation of liver tumors in CT scans. They developed a backbone network that is mostly powered by ResNeXt50 and boosted for a wider perceptual field and enhanced network depth, and boosted feature extraction efficiency without demanding more parameter adjustments. Concurrently, a Modifier was presented to improve liver tumor segmentation accuracy and augment the overall perception of the network and global grasp of the image. Hong Y et al. [32] suggested a 3D U-Net-based automatic segmentation framework with globally optimized refinement and dense connections. Initially, the probability map of the liver is fed to a deep U-Net architecture with dense connections. The probability map then acts as the opening surface and previous shape in the subsequent refinement process. The segmentation of liver tumors is predicated on a network architecture equivalent to that supporting the liver segmentation results. During the training process, an attempt is made to diminish the impacts of end-to-end tissues that have intensities and textural semblance similar to the tumor site. This can help increase segmentation accuracy. Wang J et al. [33] offered a novel network architecture to attain automatic and precise segmentation of the liver; the approach utilizes EfficientNetB4, attention gate, and residual learning approaches. The approach initially switches out the conventional convolution of the decoder for a residual block to improve the accuracy of segmentation and resolve the gradient vanishing issue. Ni Y et al. [34] presented a DA-Tran system designed to separate liver tumors from other CT segments. Initially, NC, ART, PV, and DP images are implemented for producing domain-adapted feature maps by employing a DA module. Then, 3D transformer blocks are employed to amalgamate these domain-adapted feature maps. Khoshkhabar M et al. [35] suggested a new DL approach for discriminating liver organs in CT maps and segmenting liver tumors. The presented approach consists of four layers that will exactly segment liver tumors. It is based on the LiTS17 database. In the last step, metrics are achieved by employing the LiTS17 dataset as a source. Bi R et al. [36] presented a fusion network called ResCEAttUnet, which utilizes the residual block and CE. The RMP module and a DAC component create the CE. Furthermore, the weights of the two-loss portions are distributed across training rounds by employing a hybrid loss function that combines the Tversky loss function and cross-entropy. Napte K et al. [37] suggested a liver segmentation based on the Edge Strengthening Parallel UNet (ESP-UNet) method to reject the u- and o-seg in CT scans of the liver. Moreover, this approach offered ALCD related to sequential deep CNN, which are lightweight. Evaluation criteria for the results of the ESP-UNet incorporated DCNN-based ALCD. Liu H et al. [38] presented S2DA-Net for segmentation of liver tumors. The two branches in S2DA-Net are the MAHA branch and the FSMF branch. The Fourier-based network employed by the FSMF branch acquires phase and amplitude information, which allows for the capture of more detailed and richer data without adding needless parameters. The FSMF branch enhances features, without adding needless parameters, by capturing amplitude and phase information through a Fourier-based network. The MAHA branch decreases the computational costs while improving discriminative characteristics by integrating the spatial information. A GMCA module in the decoding path creates long-term dependencies and pulls local information to improve localization capabilities by merging the features from many branches. Nallasivan G et al. [39] endeavored to estimate the performance of CNN classification of CT and Ultrasound performed for diagnosis by implementing several characteristics taken from CT and Ultrasound images. The CNN-boosted organization scheme, once advanced, revealed seven additional biased characteristics. 

### 2.1. Recent Transformer-Based Liver Tumor Segmentation Models

Recent transformer-based liver tumor segmentation models have demonstrated remarkable improvements in global context modeling and long-range dependency learning. For example, DA-Tran utilizes domain-adaptive transformers to integrate multi-phase CT information, enhancing robustness among various imaging phases. S@DA-Net integrates Fourier-based spectral learning with multi-branch attention to improve trait depiction. Gradient-Enhanced Network (GENet) enhances tumor threshold detection by learning gradient-aware features; meanwhile, hybrid CNN–Transformer models integrate local trait separation with global semantic data for better segmentation. Overall, they do not specifically serve hierarchical residual attention refinement. To resolve these benchmarks, the presented DTARNU-Net integrates the Brownian Motion-based Butterfly Optimization Algorithm (BM-BOA) with the Dense Tiered Attention Residual Module (DTARM) to refine trait illustrations, enhance trait fusion and obtain more precise live tumor segmentation.

### 2.2. Critical Comparison

The currently prevailing CNN-based methods like ResUNet enhance local trait separation but frequently encounter challenges with precision in segmenting abnormal tumor thresholds. Attention-based models, such as ERes-UNett++ and ARU-Net, improve significant trait illustration utilizing attention mechanisms. Moreover, these methods commonly achieve attention only after trait separation and do not perform adaptive calibration of traits before trait fusion. As a result, they may not completely obtain multi-scale data or precisely maintain fine tumor thresholds. Transformer-based methods such as DA-Tran and S2DA-Net effectively model long-range contextual information; however, they typically require substantially more computational resources and fuse multi-scale features directly without explicit optimization. These limitations motivate the proposed DTARNU-Net, which integrates adaptive feature optimization using BM-BOA prior to hierarchical attention refinement through DTARM.

### 2.3. Comparative Research Gap

Although recent liver tumor segmentation networks such as ResUNet [22], Eres-UNet++ [25], Rdctrans U-Net [31], ARU-Net [33], and S2DA-Net [38] integrate residual learning, attention mechanisms, or transformer-based feature extraction, several important limitations remain. First, most methods employ a single attention mechanism applied after feature extraction, which cannot progressively refine hierarchical semantic information. Second, multi-scale encoder features are directly fused without adaptive optimization, resulting in feature redundancy and loss of discriminating information. Third, existing architectures do not explicitly optimize feature complementarity before skip-connection fusion, limiting their ability to accurately segment irregular tumor boundaries and small lesions. DTARNU-Net addresses these limitations by introducing BM-BOA for adaptive feature optimization and DTARM for hierarchical residual attention refinement, thereby providing more discriminating multi-scale feature representations than existing attention-based U-Net models.

## 3. DTARNU-Net Framework

In this study, we concentrate on the precision of liver tumor segmentation utilizing CT images from the LiTs threshold dataset, which is broadly utilized in clinical imaging research. The presented work is designed to detect and accurately delineate liver tumors, enhancing the precision of liver cancer detection, treatment planning, and medical decision-making.

A. Three-level Pre-processing

We begin by introducing a three-level pre-processing approach that includes histogram equalization, noise removal, and image smoothing. This is followed by a sharpening process designed to further enhance the overall image quality.

(i) Histogram Equalization

Adaptive histogram Equalization (AHE) is a technique that improves the contrast of a CT image by adapting histogram equalization to small regions rather than the overall image. Here’s a description of how AHE works for CT images. A CT image $I\left(x,y\right)$ is divided into minimal non-overlapping regions (tiles). Assume the image is divided into M×N tiles. For an individual tile $T_{i,j}\left(k\right),$ where i and j denote the tiles indices of row and column, generate the histogram $H_{i,j}(k)$ for individual gray level k. Evaluate the cumulative distribution function (CDF) for every title as

$${CDF}_{i,j}\left(k\right)=\frac{1}{mn}\sum_{l=0}^{k}H_{i,j}(l)$$

Here, m and n are referred to as the tile dimensions $T_{i,j}$ and $H_{i,j}(l)$ is the histogram value for gray level l in the tile. The pixel intensity $I\left(x,y\right)$ of the tile is then mapped to new intensity $I^{\prime }\left(x,y\right)$ utilizing CDF:

$$I^{\prime }\left(x,y\right)=floor\left[\left(L-1\right)\times {CDF}_{i,j}(I\left(x,y\right)\right]$$

where L represents the total number of imaginable gray levels of the image and floor is the function of floor, which ensures the output will be an integer. To avoid abrupt changes at tile boundaries, the results are typically interpolated between neighboring tiles. Bilinear interpolation is commonly used for this purpose. The intensity of final pixel $I^{\prime }\left(x,y\right)$ after employing AHE to a CT image can be defined as

$$I^{\prime }\left(x,y\right)=\sum_{i=1}^{M}\sum_{j=1}^{N}\omega_{i,j}\cdot floor\left[\left(L-1\right)\times \frac{1}{mn}\sum_{l=0}^{I\left(x,y\right)}H_{i,j}(l)\right]$$

where $\omega_{i,j}$ is the weight for interpolation according to pixel proximity (x,y) to the tile center $T_{i,j}$. $H_{i,j}(l)$ is the histogram for tile $T_{i,j}$. M and N are the numbers of tiles along x and y, respectively. AHE enhances the contrast of CT images by performing histogram equalization on small tiles and interpolating between them to avoid discontinuities. This method ensures that the local contrast is enhanced, which supports underlining the significant traits in clinical images like CT scans.

(ii) Noise Reduction

The Jerman filter is specifically designed for enhancing vessel-like structures in medical images such as CT scans. It prioritizes portions which resemble tubular structures by decreasing noise. The filter’s formulation is based on the Hessian matrix, which obtains second-order derivatives of the image intensity and is sensitive to differences in curvature. Here is the basic formulation of the Jerman filter. For an image I, the Hessian matrix H is generated, as

$$H\left(r\right)=\left[\begin{array}{ccc} I_{rr}(r) & I_{rs}(r) & I_{rt}(r)\\ I_{sr}(r) & I_{ss}(r) & I_{st}(r)\\ I_{tr}(r) & I_{st}(r) & I_{tt}(r) \end{array}\right]$$

Here, $I_{rr},I_{ss}$ and $I_{tt}$, etc., denote the partial image I second-order derivation with respect to spatial coordinates r,s and t. The Hessian matrix is decomposed into eigenvalues $\Phi_{1},\Phi_{2}$ and $\Phi_{3}$ (with $\left|\Phi_{1}\right|\leq \Phi_{2}\leq \Phi_{3})$. The response of the Jerman filter $R_{J}$ is computed as

$$R_{J}=\left\{\begin{array}{c} \exp\left(-\frac{\Phi_{2}^{2}}{2c^{2}}\right)\cdot \left(1-exp\left(-\frac{\Phi_{3}^{2}}{2c^{2}}\right)\right)\\ 0,Otherwise \end{array}\right.,if\Phi_{2}<0\;and\;\Phi_{3<0}$$

where c is a constant that controls the sensitivity of the filter to the eigenvalues. The exponential terms help in reducing the response to noise (small eigenvalues) while enhancing tubular structures (significant negative eigenvalues). The final filtered image $I_{filter}$ is obtained by applying the filter response $R_{J}$ to the original image I.

(iii) Image Smoothing and Sharpening

CT image smoothing and sharpening are key techniques used to enhance image quality. Smoothing filters are used to reduce noise and soften the image, while sharpening filters enhance edges and fine details. Below are some common smoothing and sharpening filters used in CT imaging:

(a) Image Smoothing

A popular non-linear filter for image smoothing, the median filter works especially well at minimizing pepper and salt noise while maintaining edges. The way it operates is by substituting the neighborhood median value for each pixel value. For a pixel $I\left(c,d\right)$ of the image, the output of the median filter $I_{smooth}\left(c,d\right)$ is formulated as

$$I_{smooth}\left(c,d\right)=median\left\{I\left(c+i,d+e\right)|\left(i,j\right)\in N(c,d)\right\}$$

Here, $I\left(c,d\right)$ is the intensity pixel value at position (c,d) of the original image. N(c,d) is the neighbourhood about the pixel (c,d); this neighbourhood is generally described by a window (e.g. 3 × 3, 5 × 5) centered on (c,d). (i,j) are the relative pixel coordinates within the neighbourhood.

(b) Image Sharpening

High-pass filters are typically used to sharpen images by emphasizing high-frequency components, such as edges, and filtering out low-frequency components like smooth areas. However, when discussing image smoothing, we generally refer to low-pass filters rather than high-pass filters, as low-pass filters reduce high-frequency noise and result in a smoother image. That said, if you utilize a high-pass filter for sharpening, here is the equation and explanation:

A high-pass filter enhances the edges in an image by subtracting a smoothed (low-pass filtered) version of the image from the original image. Provided an image $I\left(c,d\right)$, the filtered high-pass image $I_{high-pass}(c,d)$ is calculated as

$$I_{high-pass}\left(c,d\right)=I\left(c,d\right)-I_{low-pass}\left(c,d\right)$$

where $I\left(c,d\right)$ is the original image. $I_{low-pass}\left(c,d\right)$ is the filtered version of the low pass in the original image, frequently acquired utilizing Gaussian blur or another smoothing filter.

B. Liver Tumor Segmentation

**Figure 1:**
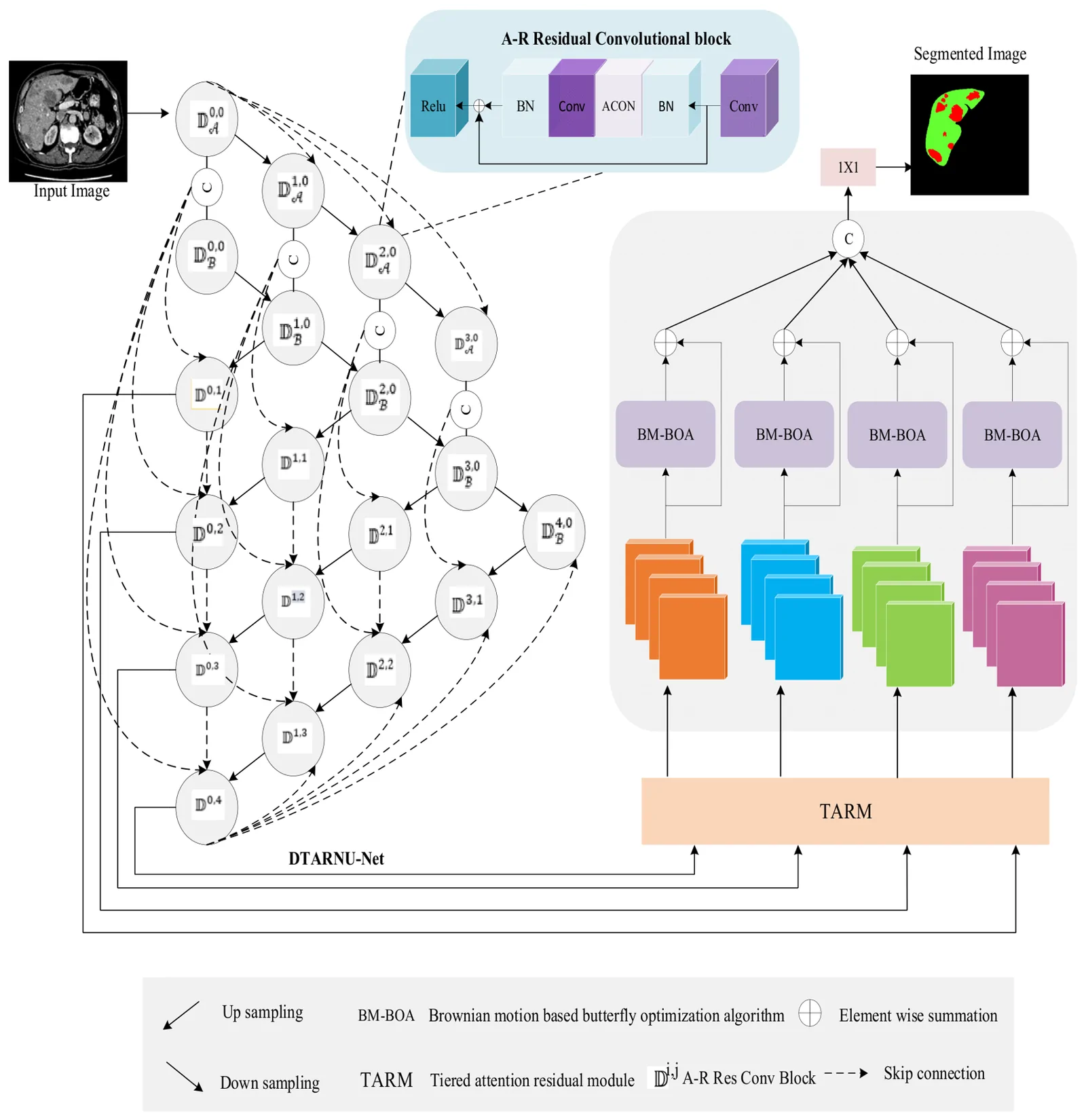
Overall architecture of DTARNU-Net—For simplicity, Figure 1 illustrates only the overall workflow of the proposed DTARNU-Net. The paired inputs III and JJJ used in the Siamese network correspond to the two parallel feature representations processed by the Siamese branches, rather than two independent CT images. Therefore, only a single CT image is shown as the network input, while the paired feature streams are generated internally within the network architecture, as described in Section 3.

We introduce DTARNU-Net, as shown in Figure 1, a model that combines a Siamese network with a nested U-Net and incorporates an A-R to enhance detection in regions with subtle changes. The model extracts diverse features, integrates them using the BM-BOA, and refines them with a Dense Tiered Attention Residual Module (DTARM) for improved liver tumor segmentation. Unlike conventional attention-based U-Net architectures, DTARNU-Net performs feature enhancement in three successive stages. Initially, BM-BOA adaptively optimizes encoder feature representations before skip-connection fusion. Subsequently, DTARM progressively refines optimized features using hierarchical residual channel-spatial attention. Finally, dense feature aggregation enables effective integration of local texture information with global semantic representations. This sequential optimization-refinement strategy constitutes the principal architectural novelty of the proposed network.

(i) Feature Extraction

The features extracted from two images are combined by applying the corresponding layer’s convolution blocks at the channel level. The features are then combined with the decoder’s features using the dense skip connection technique, as shown in Figure 1. To produce the bi-temporal characteristics $Z^{0,0}\in M^{2A\times B\times C}$, for instance, the features $Z_{I}^{0,0}\in M^{A\times B\times C}$ and $Z_{J}^{0,0}\in M^{A\times B\times C}$ from a pair of bi-temporal images I,J administered by convolutional blocks $D_{I}^{0,0}$ and $D_{J}^{0,0}$ that are initially concatenated at the channel level. Subsequently, $Z^{0,0}$ is concatenated at the channel level with the feature $Z_{J}^{1,0}\in M^{A\times B\times C}$ output from the convolutional block $D_{J}^{1,0}$ at the channel level and fed to the convolutional block $D^{0,1}$ to generate the initial stage feature $Z_{J}^{0,1}\in M^{A\times B\times C}$. Before the channel fusion operation with $Z^{0,0}$, $Z_{J}^{1,0}$ is up-sampled to observe the concatenation specification, as the pooling operation is encompassed in every stage of the encoding operation to compress the information of the features.

It is also imperative to mention that we transformed the internal organization of the convolutional block, assuming that every node $D^{e,f}$ of U-Net++ is discussed as a convolutional block. The convolutional block that we formed is a residual structure with an added ACON activation function, as shown in the figure. This redesigned convolutional block will adaptively resolve whether or not to activate particular neural nodes, subsequently enhancing the overall performance of the network. The convolutional block in question is recognizably an ACON-ReLU Residual Convolutional Block (A-R). The formula for $Z^{e,f}$ is as follows: Let $Z^{e,f}\in M^{A\times B\times C}$ be the output of A-R $D^{e,f}$; here e signifies the number of down-sampling layers, and f signifies the number of up-sampling layers.

$$Re\left(.\right)=RA(B(O\left(A\left(B\left(O\left(.\right)\right)\right)\right)+O\left(.\right))$$

$$Z^{e,f}=\left\{\begin{array}{c} M(Re\left(d^{e-1,f}\right))\\ Re([d_{A}^{e,0},d_{B}^{e,0},D(d^{e+1,f-1}\\ Re([d_{A}^{e,0},d_{B}^{e,0},[d^{e,p}{]}_{p=1}^{f-1},D(d^{e+1,f-1} \end{array}\right\}\begin{array}{c} f=0\\ f=1\\ f>1 \end{array}$$

where M signifies the down-sampling operation of max pooling, D represents the up-sampling operation of deconvolution, RA signifies Relu activation operation, B signifies the Batch Normalization operation, O represents the convolutional operation, A signifies the ACON activation operation, and [,] characterizes the channel concatenation operation for the features within it. When f > 0, the features from the neighbouring levels are merged to provide more comprehensive features with global semantic information and locally detailed information. In the encoder step, the input features are down-sampled when f = 0 in order to extract features with higher-dimensional information. In our proposed backbone network, A-R gradually increases the number of channels for the characteristics in the encoder stage. The A-R progressively reduces the number of channels in the features throughout the decoder stage, further abridging the features.

(ii) Optimized Feature Selection

BOA is a modern meta-heuristic optimization method that mimics the behavior of butterflies (BF) in searching for food and locating mates. Butterflies use chemoreceptors, sensory receptors spread across their bodies, to detect the scent of food or flowers and to find ideal mates. In the BOA, these chemoreceptors guide the movement of search agents (BF) based on the intensity of the scent. If a butterfly doesn’t detect a scent within its search area, it performs exploitation (Local Search) by moving to a randomly chosen location. However, if it senses the strongest scent as being from another butterfly, it moves toward that butterfly in an exploration (Global Search) effort. In the proposed BM-BOA, each search agent represents a candidate feature optimization vector, and the fitness function is defined by minimizing the segmentation loss (Dice loss + Binary cross-entropy loss) while maximizing feature discriminability. The BM-BOA was implemented using a population size of 30, 100 maximum iterations, a sensory modality coefficient c = 0.01, power exponent a = 0.1, and switch probability *p* = 0.8, and Brownian motion was incorporated to improve exploration and avoid premature convergence; the elephant-hearing optimization mentioned in the previous version has been removed as it is not part of the proposed BM-BOA framework. The BOA incorporates the following characteristics:

Each butterfly releases a scent that attracts others.

Butterflies move randomly toward the one emitting the strongest scent.

The intensity of the scent is influenced by the objective landscape of the problem being solved.

Update Fragrance

Each butterfly in the BOA possesses a unique scent and individuality, with the scent being a function of the stimulus intensity. The intensity of this scent will be determined by means of the following equation:

$${Frag}_{v}=S.\xi_{si}^{g}$$

${Frag}_{v}$ represents the fragrance value, which changes with each iteration and indicates how strongly other butterflies are influenced by the scent. The stimulus intensity of a butterfly is denoted as $\xi_{si}$ where g is the modality-dependent power exponent, S represents the sensory modality, and the values of g and S for the butterfly range between 0 and 1.

Butterfly Movements

During the BF movement phase, each butterfly’s position changes according to the number of iterations. As a result, every butterfly in the solution space occupies an innovative location. The fitness value of every butterfly is then evaluated and updated. Furthermore, according to Equation 6, every butterfly releases a smell from its new location. The GS phase and the Local Search LS phase are the two phases of the BOA. Butterflies in the GS phase gravitate toward those that are nearer the ideal solution, as shown by:

$$\chi_{i}^{T+1}=\chi_{i}^{T}+\left(R^{2}G^{*}-\chi_{i}^{T}\right){Frag}_{v_{i}}$$

In iteration T, where $\chi_{i}^{T}$ denotes the vector solution for the i-th butterfly, rand represents a random number in the interval [0, 1], $G^{*}$ is the optimal solution for the current iteration, and ${Frag}_{v_{i}}$ indicates the fragrance of the i-th butterfly. The LS phase is described by the following equation:

$$\chi_{v}^{T+1}=\chi_{i}^{T}+\left(R^{2}\chi_{j}^{T}-\chi_{v}^{T}\right){Frag}_{v_{i}}$$

In the solution space, $\chi_{j}^{T}$ and $\chi_{v}^{T}$ represent the j-th and v-th butterflies. R is a random number within the interval [1,2]. Random replacement can introduce limitations, such as the random replacement of the worst-performing individual and reduced exploitation, potentially leading to slower convergence. To address these issues, the BM approach is combined with the BMO. The BM is expressed as:

$${BR}_{M}=N*R_{d}\left(\cdot \right)*\omega_{|}$$

$$N=\sqrt{\frac{\vartheta }{\varphi }}$$

φ=100∗ϑ

$$z_{L}=\frac{1}{N\sqrt{2\pi }}exp\left(-\frac{{\left(dimension-agent\right)}^{2}}{2N^{2}}\right)$$

Here, ϑ represents the agent’s motion time period in seconds, and φ indicates the number of abrupt moves made by the agent over time. Integrating BM with BMO enhances the BOA. Consequently, the revised expressions for the LS and GS in the BMO are as follows:

$$\chi_{i}^{T+1}=\chi_{i}^{T}+\left({BR}_{M^{2}}G^{*}-\chi_{i}^{T}\right){Frag}_{v_{i}}$$

$$\chi_{v}^{T+1}=\chi_{i}^{T}+\left({BR}_{M^{2}}\chi_{j}^{T}-\chi_{v}^{T}\right){Frag}_{v_{i}}$$

When the termination conditions are satisfied, the butterflies stop moving, and the maximum number of repetitions acts as the ending condition. The procedure identifies the optimal solution based on fitness values. Consequently, the selected characteristics are $S\left({Frag}_{e}\right),$ represented mathematically as:

$$S\left({Frag}_{e}\right)=\left\{\sigma_{1},\sigma_{2},\sigma_{3},\ldots ,\sigma_{t}\right\}$$

Within the proposed DTARNU-Net architecture, BM-BOA is incorporated immediately after the encoder stage and before multi-level feature fusion. Rather than optimizing the network weights directly, BM-BOA operates on the extracted encoder feature maps to identify the most discriminative feature representations before they are forwarded through the skip connections. Each butterfly represents a candidate feature-selection solution, while the optimization objective is defined by maximizing segmentation quality through improved feature discrimination and boundary localization. The refined trait illustrations are additionally transmitted to the DTARM module, where hierarchical attention refinement is conducted previous to decoding. This optimization-before-fusion strategy enables the decoder to receive more informative semantic representations, thereby improving segmentation accuracy.

**Figure 2:**
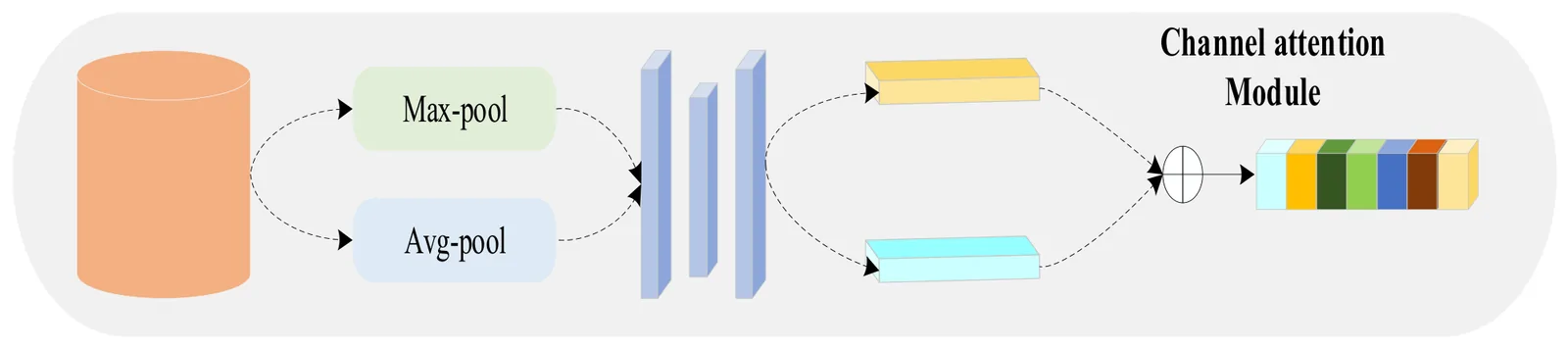
CAM workflow.

As opposed to the conventional U-Net architectures, which straightly concatenate encoder and decoder traits, BM-BOA executes adaptive optimization of encoder features illustrating previous feature fusions. Specifically, BM-BOA optimizes the feature activation coefficients corresponding to different semantic channels extracted from the encoder. The optimization objective simultaneously maximizes inter-class feature separability and minimizes redundant feature responses, thereby improving feature complementarity, see Figure 2. The incorporation of Brownian motion enhances exploration by preventing premature convergence to local optima and maintaining population diversity throughout the optimization process. Consequently, the optimized feature maps provide more discriminative semantic information for the subsequent DTARM refinement stage, resulting in superior boundary localization and improved segmentation of small and irregular liver tumors.

B. Segmentation

We incorporate CBAM to improve DTARM, which syndicates a CAM and a SAM. The prime function of the CAM module is to utilize the relationship among feature channels to generate a channel attention map. The channel attention map will be implemented to recalibrate the corresponding feature weights at the channel level. The process is outlined in the figure, which depicts CAM initially gathering spatial information on the input features $Z\in M^{A\times B\times C}$ by applying adaptive max pooling and adaptive average pooling, both with an output scale of 1. This produces two C-dimensional pooling features, $Z_{max1}\in M^{A\times 1\times 1}$ and $Z_{avg1}\in M^{A\times 1\times 1}$, that are fed beforehand to a common MLP. After element summation and sigmoid activation, the two features are output to create the crucial channel attention map $R_{T}\in M^{A\times 1\times 1}$. The following equation is the rapid calculation for the CAM:

$$R_{T}\left(Z\right)=\varphi (MLP\left(Avgpool\left(Z\right)\right)\pm MLP\left(Maxpool\left(Z\right)\right))$$

**Figure 3:**
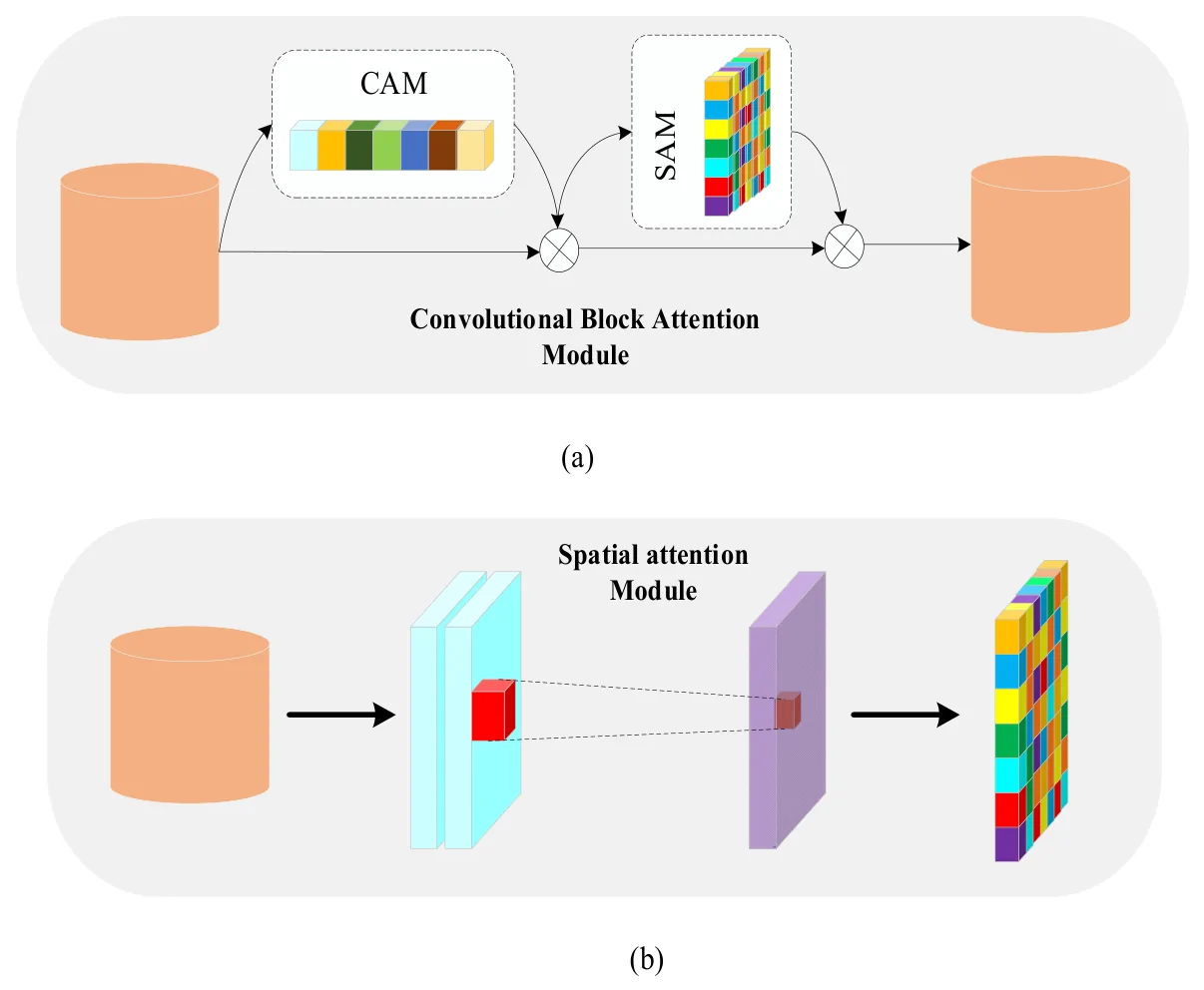
(**a**) Convolutional block–attention module and (**b**) spatial attention module.

Here, Z characterizes the input features, φ signifies the sigmoid activation function, ± represents the element-wise addition, and the MLP weights are shared. By multiplying the channel attention map by the associated features, it is possible to improve the features at the channel level as well as achieve the goals of improving relevant channels, eliminating irrelevant channels, and obtaining globally optimized features at the channel level. To acquire the desired spatial attention map $R_{Q}\in M^{1\times B\times C}$, SAM initially performs maximum pooling and average pooling on the input features $Z\prime \in M^{A\times B\times C}$ along the channel direction. This operation produces two two-dimensional features. $Z_{max2}\in M^{1\times B\times C}$ and $Z_{avg2}\in M^{1\times B\times C}$. These two feature maps are then fed into a layer of convolution, following concatenation at the channel level, see Figure 3. Finally, the sigmoid activation function stimulates these two feature maps. The SAM is in core as envisioned as follows:

$$R_{Q}\left(Z^{\prime }\right)=\varphi (Z^{7\times 7}\left([Avgpool\left(Z\prime \right)\right)*\left(Maxpool\left(Z\prime \right)\right))$$

Here, $Z^{\prime }$ signifies the input features, φ represents the sigmoid activation function, ∗ signifies the channel concatenation, and $Z^{7\times 7}$ represents a convolution operation with a convolution kernel size of 7 × 7. By multiplying the 2D features generated by the spatial attention module by the pertinent qualities, the spatial layer of the final output feature map can be adaptively changed to challenge the given value. This can withstand the transformation of each pixel position’s weight value.

Figure 3 demonstrates how CBAM, composed of the CAM followed by SAM, effectively suppresses or ignores irrelevant channel and spatial location information while guiding the network to focus on the most important features. By integrating spatial position data, the model’s feature representation is enhanced at critical points, reducing the negative impact of unnecessary information on decision-making. This attention-based approach allows the network to independently optimize the input feature representation at both the channel and spatial levels. The computation of CBAM is detailed as follows:

$$CBAM\left(Z\right)=R_{Q}\left(R_{T}\left(Z\right)⨂Z\right)⨂\left(R_{T}\left(Z\right)⊗Z\right)$$

Here, $\left(Z\right)$ represents both of the input features and ⨂ is the element-wise multiplication operation. The corresponding channel attention map and spatial attention map generated from the features $\left(Z\right)$ are denoted by $R_{T}\left(Z\right)$ and $R_{Q}\left(Z\right)$, respectively. Let $Z\in R^{C\times H\times W}$, where $Z_{i}^{Q}\in R^{C\times H\times W}$ represents the channel-level feature mapping subsets obtained by equally dividing the input feature map $i\in \left\{1,2,\ldots ,X\right\}$ with C/X subsets within the DTARM framework. Each subset $Z_{i}^{Q}$ has the same spatial dimensions as $\left(Z\right)$, but with reduced channels, now C/X. We apply the CBAM method to each $Z_{i}^{Q}$, incorporating a residual structure to ensure that the valuable information in the original features is not simply discarded. After processing, the output for each $Z_{i}^{Q}$ is denoted as $Y_{i}^{Q}\in R^{C/X\times H\times W}$, where $i\in \left\{1,2,\ldots ,X\right\}$. Each subset of feature mappings, except for $Z_{1}^{Q}$, is concatenated with the processed data from the previous layer before being processed by CBAM. After enhancing all feature mapping subsets, we concatenate the X output features, with the final output represented as $Z_{R}\in R^{C\times H\times W}$. The precise formulation for DTARM is as follows:

$$Y_{i}^{Q}=\left\{\begin{array}{c} CBAM\left(Z_{i}^{Q}\right)⊕Z_{i}^{Q}i=1\\ CBAM\left(Z_{i}^{Q}⨁\left(CBAM\left(Z_{i-1}^{Q}\right)⨁Z_{i-1}^{Q}\right)\right)⨁Z_{i}^{Q}i>1 \end{array}\right.$$

$$Z_{R}=\left[y_{1}^{Q},y_{2}^{Q},\ldots ,y_{i}^{Q}\right]i\in \left\{1,2,\ldots ,X\right\}$$

Here, [⊕] denotes the concatenation of feature map subsets at the channel level, ⨁ represents element-wise summation, and $CBAM\left(\cdot \right)$ refers to the sequential channel and spatial enhancement of features. Utilizing this method, the output feature $Z_{R}$ of DTARM efficiently integrates the optimized trait data. Experimental testing has shown that DTARM performs optimally with three branches, or X=3. As opposed to the conventional attention modules, such as CBAM and SE, which produce a single global attention map for the whole trait, DTSRM here separates the trait channels into various hierarchical groups. Each group is autonomously preserved utilizing residual attention, and the improved traits are developmentally collaborated through dense links. This hierarchical streamline maintains fine tumor threshold information, decreases data loss, and enhances segmentation accuracy compared with recurrent world attention operation.

## 4. Experimental Results

To examine the efficiency of the presented DTARNU-Net, ablation learning tests were executed to detect the support of each separate module. The segmentation implementation of the presented model was compared with multiple state-of-the-art methods. Both qualitative and quantitative methods were undertaken to examine segmentation precision and to underline the benefits of the presented method.

### 4.1. Dataset Description

All experiments in this study were executed utilizing the Liver Tumor Segmentation (LiTS) boundary dataset. The dataset consists of contrast-enhanced abdominal CT scans with specialist-annotated liver and tumor masks. LiTS was chosen, as it involves a large number of diverse medical cases with important differences in tumor size, shape, location, and intensity, making it capable of testing the efficiency of deep learning-based liver tumor segmentation models. The pre-processing, validation, testing, ablation studies, and facilitating contrast were carried out utilizing only the LiTS dataset. The 3D-IRCADb-01 dataset is highlighted only in the Section 2 for purposes of contrast with earlier studies and was not utilized for training or testing the proposed DTARNU-Net.

### 4.2. Liver Tumor Segmentation (LiTS) Dataset

LiTS is a public dataset broadly utilized for automatic liver and liver tumor segmentation from contrast-enhanced CT images. It was implemented through the IEEE ISBI/MICCAI LiTS challenge and consists of 201 abdominal CT scans, including 131 comment training scans and 70 hidden test scans gathered from seven international medical centers. The dataset supports specialist comments on pixel-level masks for three classes: background, liver, and liver tumor. It involves large differences in tumor size, shape, number, location, intensity, and contrast improvement, involving both hypodense and hyperdense lesions. Subsequently, the CT scans present the required variations in liver anatomy and imaging protocols among patients. These traits make the LiTS dataset a challenging and reliable threshold for examining the precision, robustness, and normalization capability of liver tumor segmentation models.

### 4.3. 3D-IRCADb-01 Dataset

The 3D-IRCADb-01 dataset is a publicly accessible dataset improved by the Institut de Recherche contre less Concers de I’Appareil Digestif (IRCAD), France, for liver segmentation and surgical planning research. It consists of 20 contrast-enhanced abdominal CT scans gathered from 10 male and 10 female patients, with approximately 75% of the cases consisting of one or more liver tumors of various sizes and placements. Every patient dataset involves the real DICOM CT images, labeled anatomical structures, binary segmentation masks, and three-dimensional (3D) surface mesh (VTK) files. The labels occupy several structures, involving the liver, liver tumors, portal vein, hepatic vein, vena cava, lungs, bones, skin, and other abdominal organs. The dataset also consists of broad liver anatomies, tumor spread, and imaging artifacts, making it a valuable benchmark for examining the reliability and normalization of liver and multi-organ segmentation algorithms. Overall, this dataset is broadly utilized in research; it was not utilized in the experiments proposed in this study.

A. Evaluation Index

**Figure 4:**
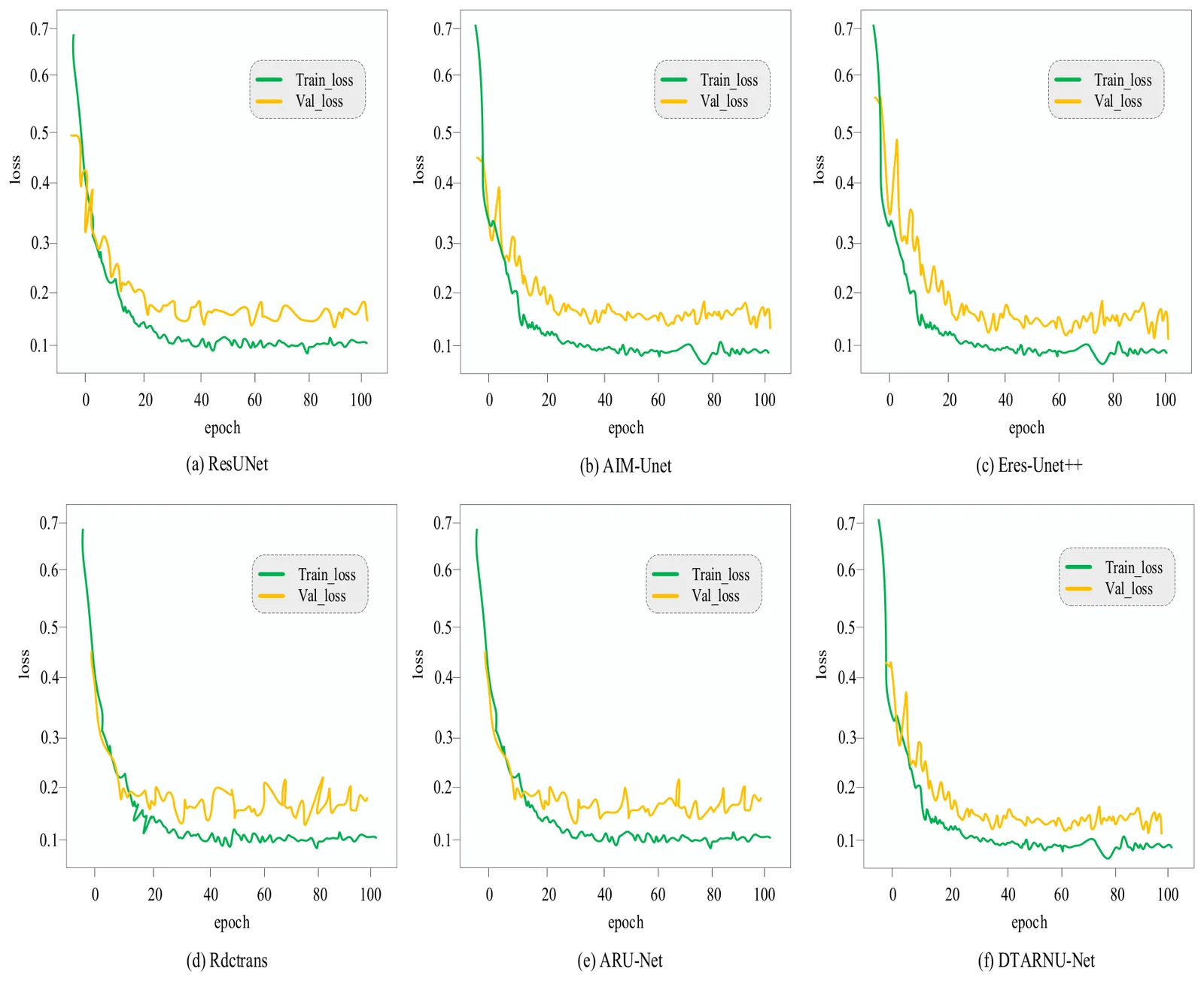
Performance analysis of train loss and validation loss.

A confusion matrix is a broadly utilized tool for examining the efficiency of semantic segmentation models. It contrasts the predicted segmentation with the manually labeled reference standard at the pixel level. Depending on the confusion matrix, multiple evaluation metrics can be calibrated to assess the precision and reliability of the model. These metrics are computed utilizing a fixed decision boundary. In the confusion matrix, true negative (TN) designates background pixels that are rightly classified as background, while true positive (TP) designates target pixels that are rightly classified as target areas. False positive (FP) designates background pixels that are wrongly classified as target pixels, while false negative (FN) designates target pixels that are wrongly classified as background. These designations are used to determine indices that describe the calibration effectiveness, metrics like Precision, Recall, F1-Score, Dice coefficient, and Intersection Over Union (IoU). Training loss and validation loss are given in Figure 4.

The Dice coefficient is a similarity metric broadly utilized for examining the efficiency of image segmentation models. It assigns a value to the intersection between the predicted segmentation and the target label segmentation. A Dice coefficient value closer to 1 highlights a higher degree of relation and better segmentation precision, whereas a value close to 0 highlights poor intersection. The Dice coefficient is calibrated utilizing the following formula:

$$\in_{DC}=\frac{2\left|X\cap Y\right|}{\left|X\right|+\left|Y\right|}=\frac{2\nabla_{TP}}{2\nabla_{TP}+\nabla_{FP}+\nabla_{FN}}$$

Precision is the value of the fraction of correctly classified pixels to the total number of pixels in the image. Thus, it highlights the overall efficiency of the segmentation model. A higher Precision value demonstrates better pixel classification and more robust segmentation outcomes. The Precision is calibrated utilizing the following formula:

$$\in_{ACC}=\frac{\nabla_{TP}+\nabla_{TN}}{\nabla_{TP}+\nabla_{TN}+\nabla_{FP}+\nabla_{FN}}$$

A necessary metric for retrieving the accuracy of image segmentation is mIoU. Use the following formula to get the IoU value for each category:

$$\in_{MioU}=\frac{1}{k+1}\sum_{i=0}^{k}\frac{\rho_{ii}}{\sum_{j=0}^{k}\rho_{ij}-\rho_{ii}}=\frac{1}{2}\left(\frac{\nabla_{TP}}{\nabla_{TP}+\nabla_{TN}+\nabla_{FP}}+\frac{\nabla_{TN}}{\nabla_{TN}+\nabla_{FP}+\nabla_{FN}}\right)$$

FwIoU aggregates the IoU values of each category by multiplying them with weights determined by the frequency of each category. The formula for calculating FwIoU is as follows:

$$\in_{FwioU}=\frac{\nabla_{TP}+\nabla_{FN}}{\nabla_{TP}+\nabla_{TN}+\nabla_{FP}+\nabla_{FN}}*\frac{\nabla_{TP}}{\nabla_{TP}+\nabla_{FP}+\nabla_{FN}}$$

Precision measures accuracy by indicating the percentage of pixels appropriately classified as positive areas of interest. Here is the formula used to determine Precision:

$$\in_{Pre}=\frac{\nabla_{TP}}{\nabla_{TP}+\nabla_{FP}}$$

Recall, also called the true positive rate, represents the percentage of accurately segmented pixels among all genuinely positive examples. Here is the formula used to determine Precision:

$$\in_{Re}=\frac{\nabla_{TP}}{\nabla_{TP}+\nabla_{FN}}$$

F1-Score is the harmonic mean of Recall and Precision, with a value range of [0, 1]. A value closer to 1 indicates improved segmentation performance. The formula for calculating the F1-Score is as follows:

$$\in_{F1}=\frac{2\in_{Pre}*\in_{Re}}{\in_{Pre}+\in_{Re}}$$

IoU ratio measures the overlap between the label and predicted value relative to their union. The IoU value increases as the overlap between the label and the prediction grows. An IoU ratio of 1, indicating complete overlap, represents the optimal scenario. The formula for calculating IoU is as follows:

$$\in_{IoU}=\frac{{Detection}_{result}\cap {Ground}_{truth}}{{Detection}_{result}\cup {Ground}_{truth}}$$

B. Implementation Details

This study was conducted using the Windows 10 operating system and the PyTorch framework. The training and validation processes were performed on an NVIDIA GeForce RTX 2165 GPU and an Intel Core i5-7600 CPU. Endeavoring to ensure a fair comparison, all methods were tested under the same conditions. The dataset was randomly split into training, validation, and test sets, with a 9:2:2 ratio. The LiTS dataset was partitioned using a patient-wise (CT volume-level) strategy to prevent data leakage between the training, validation, and testing sets. The complete dataset consisting of 201 CT volumes was divided according to a 9:2:2 ratio, resulting in 139 CT volumes for training, 31 CT volumes for validation, and 31 CT volumes for testing. All image slices belonging to a particular CT volume were assigned exclusively to a single subset, ensuring that slices from the same patient did not appear simultaneously in different subsets. This patient-wise partitioning provides a fair and unbiased evaluation of the proposed DTARNU-Net by assessing its generalization ability on previously unseen patient data. Given the limited availability of medical images, data augmentation methods such as rotation and translation are employed to reduce overfitting. To enhance the presented DTARNU-Net, the Adam optimizer was utilized rather than the currently prevailing Stochastic Gradient Descent (SGD) due to its quick convergence and superior effectiveness for large-scale deep learning models. The accelerator parameters were cluster to β_1_ = 0.9, β_2_ = 0.999, ε = 10^−8^, and weight decay = 1 × 10^−5^. The early learning rate was 1 × 10−^2^ for the first 50 epochs and slowly decreased to 1 × 10^−5^ over the remaining epochs, utilizing a cosine annealing learning rate scheduler. The network was developed with a batch size of 4 for 100 epochs, and initial stopping with a patience of 15 epochs was performed, depending on the validation loss, to avoid overfitting. Training effectively utilized mixed-precision computation to decrease GPU memory usage and enhance computational effectiveness. The model with the lowest validation loss was selected for final examination, and test-time augmentation was not employed. To ensure reproducibility, all experimental procedures were repeated five times utilizing various random seed values, and the average effectiveness was documented. For a correct comparison, all baseline models involving ResUNet [22], AIM-Unet [23], Eres-UNet++ [25], Rdctrans U-Net [31], and ARU-Net [33] were employed under the same experimental setting. All methods utilized the detected LiTS dataset split, pre-processing pipeline, data augmentation strategy, optimizer settings, learning rate schedule, batch size, training epochs, and hardware configuration. This consolidated protocol confirms that the efficiency variations result from the network frameworks, rather than variations in the training environment.

The presented method was examined solely on the LiTS threshold dataset, which consists of contrast-enhanced abdominal CT scans with specialist-labeled liver and tumor masks. All CT slices were resized to 256 × 256 pixels and fragmented at the patient level into 69% training, 15.5% validation, and 15.5 testing (9:2:2 ratio) to avoid data leakage. The LiTs dataset was utilized for model training, validation, hyperparameter tuning, ablation studies, statistical analysis, and comparative examination. Overall, the 3D-IRCADb-01 dataset is described in the Section 2; it was not utilized in any experiments. For previous training, every CT image was organized utilizing the presented three-level pre-processing pipeline. First, adaptive histogram equalization (AHE) was employed to enhance local image contrast. Next, the Jerman filter was utilized to decrease noise while retaining vessel-like structures. Lastly, median filtering and high-pass sharpening were effected to reject residual noise and improve tumor thresholds. All pixel intensities were generalized to the range [0, 1] prior to use. To enhance model reliability and decrease overfitting, data augmentation methods involving horizontal flipping, vertical flipping, random rotation (±15°), elastic deformation, and random scaling were implemented during training. The network weights were defined beforehand utilizing the default PyTorch 2.10 method, and all experiments were conducted in the PyTorch architecture under the described software and hardware configurations to ensure reproducibility. The network parameters were streamlined utilizing a hybrid loss function that combines Dice loss and binary cross-entropy (BCE) loss. Dice loss enhances the integration between the forecast and ground truth segmentation masks, while BCE loss improves pixel-level classification precision. The integrated loss function offers a more precise and robust liver tumor segmentation. The total loss is computed as

$$L_{total}=0.5L_{Dice}+0.5L_{BCE}$$

This blending efficiently maintains region intersection and threshold preservation, resulting in more reliable liver tumor segmentation.

Computational Complexity Analysis

To evaluate the computational efficiency of the presented DTARNU-Net, a detailed analysis was conducted by evaluating the number of trainable parameters and floating-point operations (FLOPs), the GPU memory consumption, and the inference time under identical experimental conditions. DTARNU-Net consists of 34.82 million trainable parameters and requires 52.61 GFLOPs for processing a single CT volume. During inference, the model is estimated to utilize 4.38 GB of GPU memory on an NVIDIA GeForce RTX 2165 GPU. The average inference time of DTARNU-Net was 0.183 s per CT slice (or 7.84 s per CT volume), illustrating its utility for practical medical applications. Compared with ResUNet, which has 31.45 million parameters and needs 48.92 GFLOPs, DTARNU-Net presents only a moderate increase in computational complexity while offering substantially enhanced segmentation performance. In the same way, in contrast with Eres-UNet++, which required 55.73 GFLOPs and 4.92 GB GPU memory, the presented network obtains better segmentation precision with lower computational burden. Although the integration of BM-BOA and DTARM slightly increases the model complexity, BM-BOA maintains only the encoder traits demonstration compared to the entire network, thereby thresholding the extra computational cost. Further, DTARM improves trait learning through hierarchical attention fine-tuning, without increasing the number of parameters. Overall, DTARNU-Net obtains efficient stability between segmentation accuracy and computational effectiveness, supplying higher performance with only a modest increase in the use of computational resources, in contrast with the prevailing state-of-the-art methods.

C. Ablation Study

To examine the efficiency of every presented element, an ablation study was executed by slowly including individual modules with the baseline UNet++ framework, with the same experimental setup. The baseline UNet++ obtained a Dice score of 92.81%, IoU of 90.46%, and Accuracy of 94.32%, highlighting that conventional trait extraction alone is not enough for precisely segmenting small and abnormal liver tumors. After integration with the presented tri-level pre-processing module, the Dice score increased to 93.88%, IoU was enhanced, rising to 91.54%, and Accuracy improved to 95.13%. These enhancements illustrate that adaptive histogram equalization, Jerman filtering, median smoothing, and image sharpening effectively improve tumor visibility, decrease image noise, and enhance segmentation efficiency. This pre-processing stage produced an absolute improvement of 1.07% in Dice and 1.08% in IoU compared with the baseline. The proposed three-level pre-processing pipeline was designed to improve image quality while preserving diagnostically relevant anatomical structures before feature extraction. Histogram equalization enhances global contrast, thereby improving the visibility of low-contrast liver tumors. The denoising stage suppresses high-frequency imaging noise while preserving major structural information, using edge-preserving filtering. Subsequently, smoothing is applied to reduce minor intensity fluctuations and improve the stability of feature learning during network training. Finally, image sharpening enhances local edge contrast to improve tumor boundary delineation before segmentation. Although aggressive pre-processing may potentially introduce artificial edges or over-enhancement, the adopted pre-processing parameters were selected conservatively to minimize such effects. Furthermore, the sequential combination of denoising before sharpening reduces the possibility of noise amplification during edge enhancement. Visual inspection of the pre-processed CT images confirmed that the proposed pipeline preserved clinically relevant tumor boundaries without introducing noticeable artifacts. Subsequently, replacing the conventional convolution block with the proposed A-R residual block further improved feature extraction, resulting in a Dice score of 94.95%, IoU of 92.71%, and Accuracy of 95.98%. The addition of the A-R block enhanced the Dice score by approximately 1.07%, indicating that the remaining learning improves local trait separation and retains fine tumor threshold information. Integrating the presented BM-BOA further improved the Dice score to 95.96%, IoU to 94.15%, and Accuracy to 96.54% by adaptively optimizing encoder traits, illustrating prior trait fusion. Compared with the previous configuration, BM-BOA provides an additional 1.01% Dice improvement and 1.44% IoU enhancement while decreasing redundant feature responses. Finally, integrating the complete DTARM obtained the best performance, obtaining a Dice score of 97.00%, IoU of 95.82%, and Accuracy of 96.78%. DTARM alone contributed a further 1.04% increase in Dice and 1.67% enhancement in IoU through hierarchical residual attention refinement and dense feature aggregation. In conclusion, the complete DTARNU-Net obtained an absolute improvement of 4.19% in Dice, 5.36% in IoU, and 2.46% in Accuracy compared with the baseline UNet++. The consistent advances in development after the addition of each module suggest that each presented component contributes individually and complements the others. These conclusions illustrate that the integrated use of the three-level pre-processing pipeline, A-R block, BM-BOA, and DTARM progressively enhances trait quality, trait efficiency, and attention-based feature refinement, resulting in more precise liver tumor segmentation.

D. Quantitative Analysis

**Figure 5:**
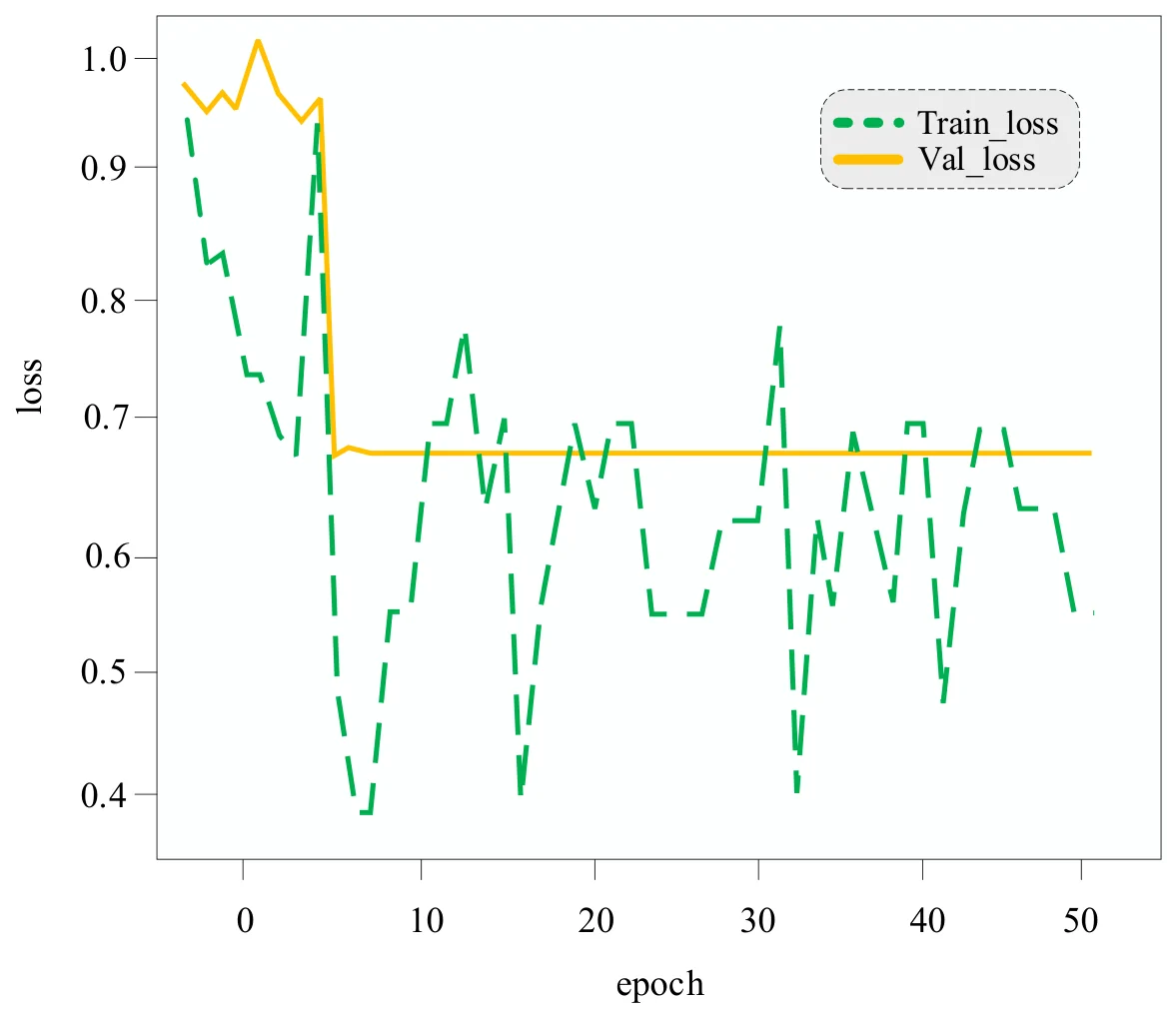
Loss analysis.

**Figure 6:**
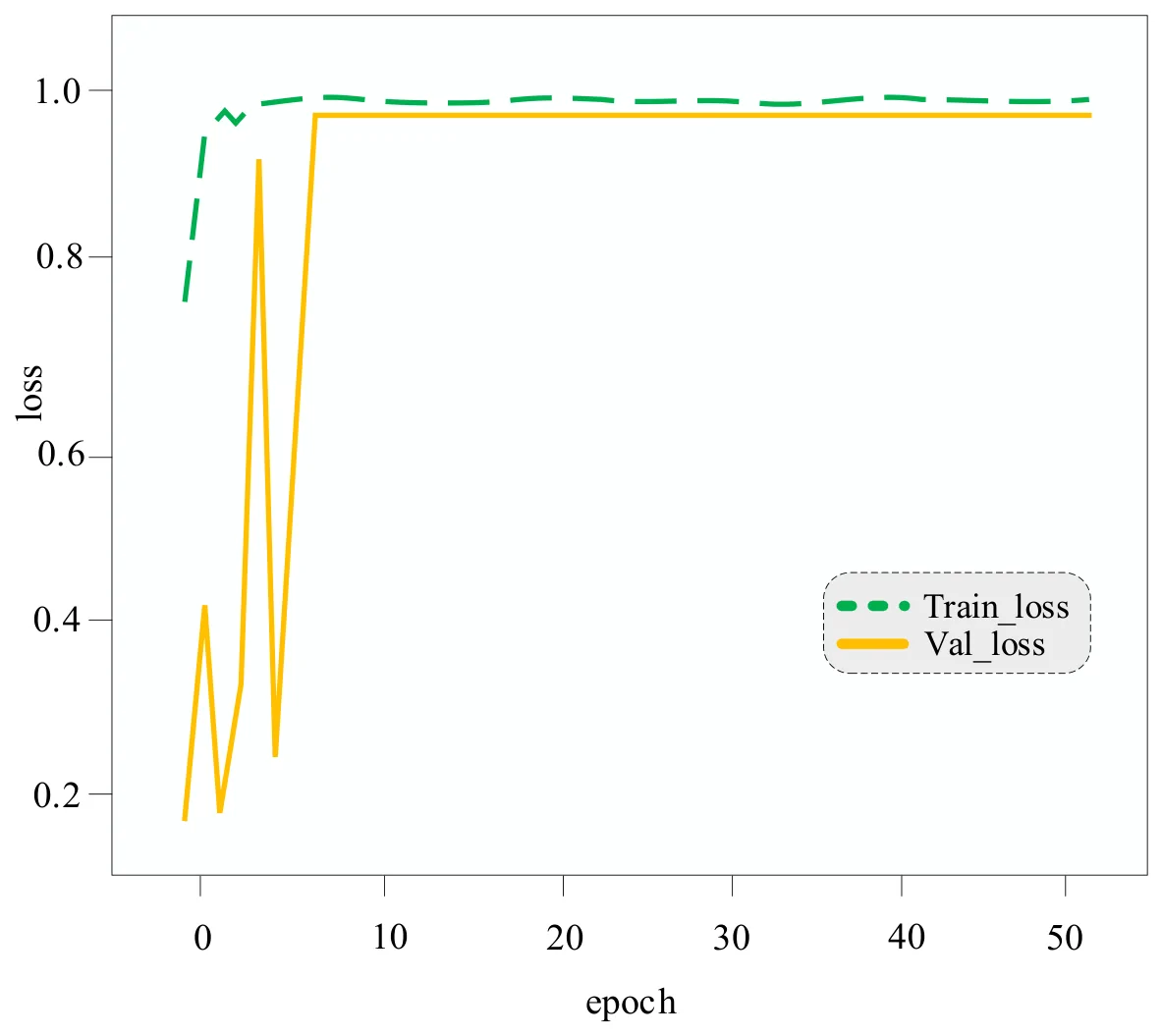
Loss analysis.

The segmentation model is considered well-trained when the loss function decreases and converges toward zero. The presented model was examined on the test set, and its segmentation performance was evaluated utilizing the predicted images. The quantitative comparison is proposed in Table 1, where method 5 (DTARNU-Net) surpasses the baseline U-Net and all other contrasted methods across all evaluation metrics. Figure 5 and Figure 6 represent the loss analysis. In the comparison among them, method 5 obtains the lowest loss values and the fastest convergence during training. This also illustrates stable training and validation performance, highlighting better learning ability and normalization. The outcome conclusively indicates that DTARNU-Net is the most efficient segmentation model across the evaluated methods.

The U-Net series is one of the deep learning models broadly utilized for biomedical image segmentation. It consists of an encoder and a decoder linked by skip connections that support the maintenance of spatial data and decrease problems like gradient vanishing and network degradation. Moreover, the efficiency of U-Net may be reduced in challenging segmentation tasks, specifically, when tumor limitations are unclear or irregular, making precise segmentation more difficult. To enhance network flexibility, the recently introduced UNet++ incorporates deep supervision and the DenseNet structure. This enhancement significantly improves training accuracy, particularly for tumors with substantial shape changes and indistinct limitations. A comparison between methods 1 and 2 demonstrates that UNet++ consistently outperforms U-Net in various metrics. Therefore, this article adopts UNet++ as the baseline model, from among ResUNet [22], AIM-Unet [23], Eres-UNet++ [25], Rdctrans u-net [31], and ARU-Net [33].

(i) Performance Analysis of Liver Segmentation

**Figure 7:**
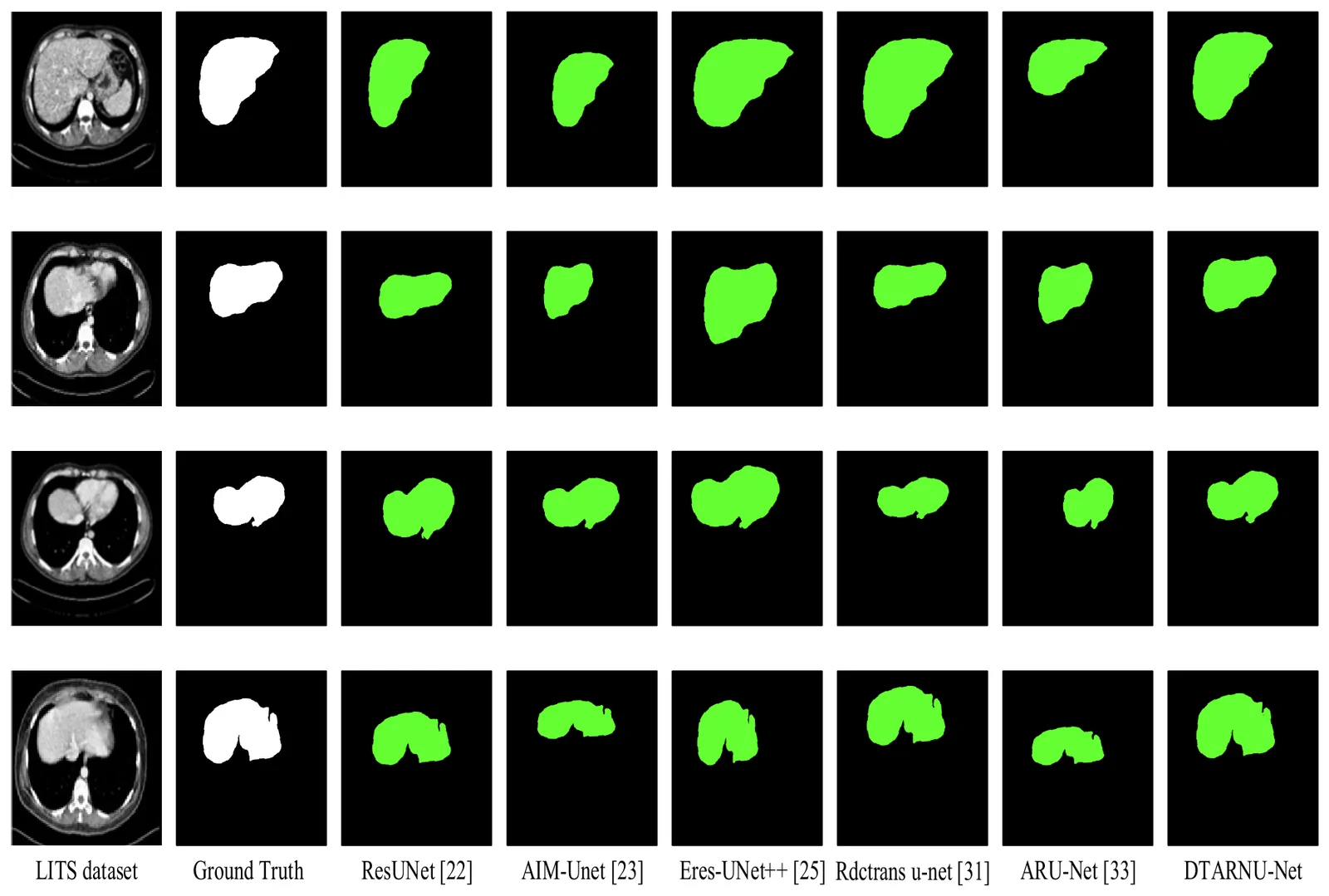
Liver segmentation performance analysis.

Ablation experiments were used to evaluate U-Net and the various network modules proposed in this paper. The effectiveness of these networks on liver segmentation is assessed using four indicators: $\in_{ACC},\in_{IoU},\in_{F1}$ and $\in_{Re}$. The consequences, revealed in Table 2, indicate which presented method outperforms other approaches in segmentation performance. Figure 7 presents four liver segmentation scenarios to provide a clearer view of the qualitative results. Column one displays the CT scan, column two shows the manual annotation by a doctor, column three depicts the segmentation result from U-Net, and column four shows the result from UNet++. Column five illustrates the segmentation outcome with the ECA module added to UNet++. The prevailing liver segmentation models have multiple limitations. ResUNet efficiently segmented the liver and liver tumors from the CT images but may lose fine threshold information due to the fact that its residual links can smooth significant features. AIM-UNet obtains good performance; its challenging framework and reliance on particular datasets, however, may decrease its capability to generalize and increase the risk of overfitting. ERes-UNet++ joins channel attention with the Res_UNet++ backbone, but its efficiency may be reduced when segmenting livers with large differences in shape or size, or irregular anatomy. Rdctrans U-Net collaborates convolutional and transformer-based traits, offering higher levels of adaptivity, but its hybrid framework can make it harder to balance variation among network components, leading to variable segmentation efficiency among diverse CT datasets.

The comparative evaluation assessed efficiency under significant experimental rigor to ensure a fair and unbiased comparison of all segmentation models. All baseline methods were trained and tested utilizing the same LiTS dataset split, pre-processing pipeline, data augmentation strategy, optimizer, learning rate schedule, batch size, and training epochs as the presented DTARNU-Net. The segmentation efficiency was examined utilizing the Dice similarity coefficient (DSC), Intersection Over Union (IoU), Accuracy, Precision, and Recall. As shown in the experimental results, the proposed DTARNU-Net achieved a Dice score of 97.00 ± 0.18%, IoU of 95.82 ± 0.21%, Accuracy of 96.78 ± 0.15%, Precision of 96.43 ± 0.24%, and Recall of 97.15 ± 0.19%. Compared with the strongest baseline, DTARNU-Net enhances the Dice score by 1.24%, IoU by 1.08%, and Accuracy by 0.86%, highlighting consistent effective enhancements among overall evaluation metrics. The higher Precision highlights the fewer false-positive forecasts; meanwhile, the enhanced Recall represents better detection of tumor regions, with fewer missed lesions. The improved Dice score and IoU also indicate better agreement between the forecast segmentation and the specialist-labeled reference standard. These outcomes illustrate that DTARNU-Net offers more precise, reliable, and robust liver tumor segmentation than the prevailing state-of-the-art methods under common experimental situations.

(ii) Performance Analysis of Liver Tumor Segmentation

CT scans can differentiate between liver cancer and healthy liver tissue by measuring CT values. However, segmenting the tumor area on a CT image is challenging due to the tumor’s potentially irregular shape, similar density to adjacent tissues, and uneven density, making it difficult to identify clear boundaries. Consequently, tumor segmentation accuracy is generally lower, compared to that of liver segmentation. In an attempt to assess the effectiveness of the presented network for tumor region segmentation, ablation experiments were conducted using the U-Net as a baseline, comparing its performance against various modules of the network. The metrics used for evaluation were $\in_{ACC},\in_{IoU},\in_{F1}$ and $\in_{Re}$, which are similar to those used for liver segmentation. The experimental results for five methods, namely, method 1 (ResUNet), method 2 (AIM-Unet), method 3 (Eres-UNet++), method 4 (Rdctrans U-Net), and method 5 (ARU-Net), with the proposed DTRANU-Net are presented in Table 3. When compared to U-Net, UNet++ shows an average improvement of 4.2% in Accuracy, 5.34% in IoU, 2.82% in F1-Score, and 2.64% in Recall.

**Figure 8:**
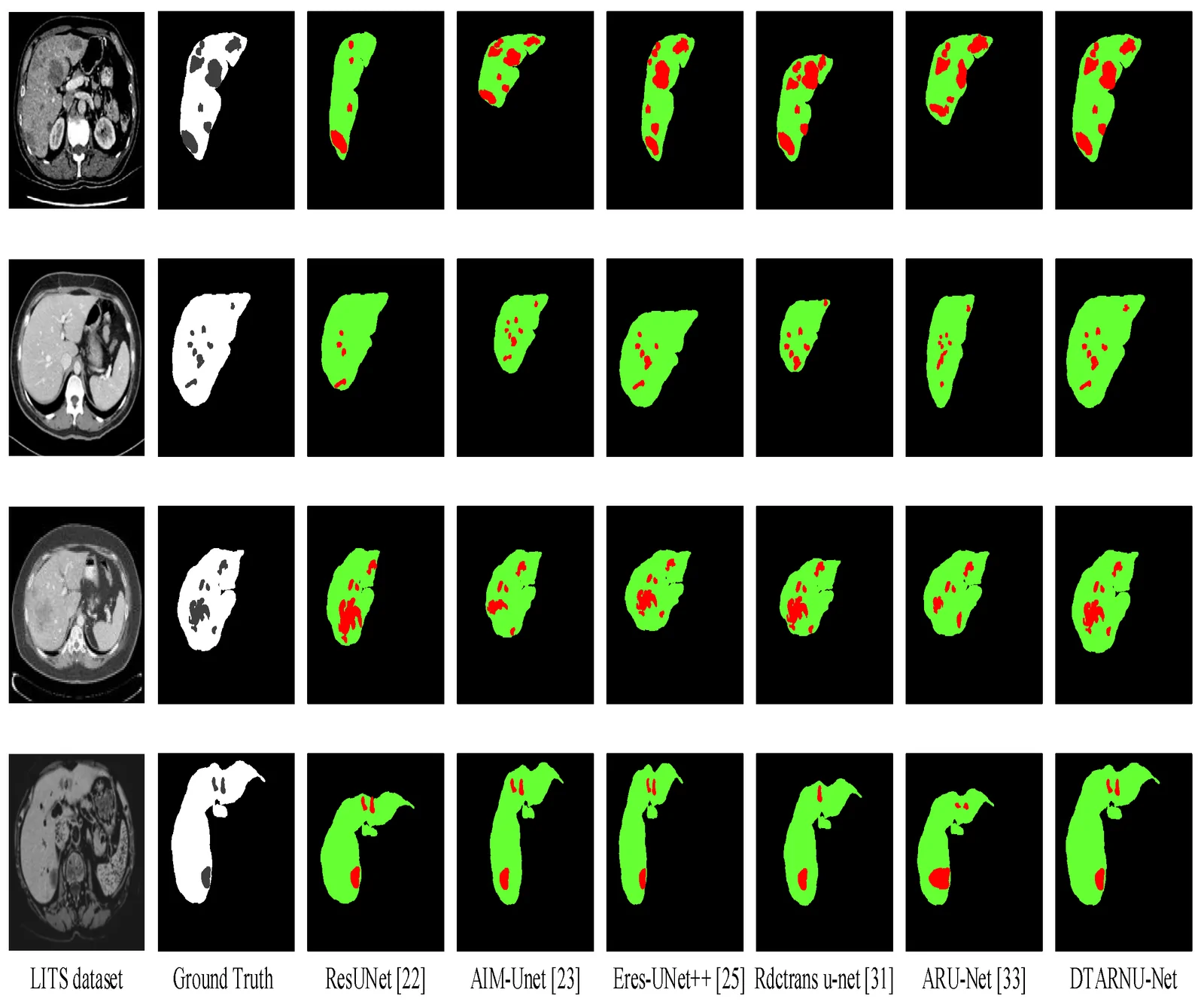
Liver tumor segmentation performance analysis.

Additionally, the proposed DTRANU-Net method outperforms the original UNet++ by improving Accuracy. In medical image segmentation, higher Recall is particularly important, as it more accurately identifies lesion regions, reducing the risk of missed diagnoses. These results demonstrate that the proposed network effectively segments tumor regions. Figure 8 illustrates four sets of tumor segmentation results, where the liver segmentation is highlighted in red and the tumor is represented as an irregular green area. Column a shows the CT image, column b presents the manual annotation, and columns c through g display the segmentation results of ResUnet, AIM-Unet, Eres-UNet++, Rdctrans U-Net, and ARU-Net, as well as the proposed DTRANU-Net, respectively. The results in column g, corresponding to the DTRANU-Net algorithm, indicate which proposed system successfully segments the tumor region. In contrast, the U-Net and UNet++ networks struggle with smoothly segmenting the edges of tumors, leading to challenges in accurately segmenting small and irregular lesion areas.

## 5. Discussion

Compared with existing attention-based U-Net architectures, DTARNU-Net introduces two complementary innovations that jointly improve segmentation performance. BM-BOA refines encoder traits before character fusion, while the Dense Tiered Attention Residual Module (DTARM) successively optimizes these traits utilizing hierarchical residual attention. As opposed to conventional models that implement attention only after trait separation, DTARNU-Net integrates trait refinement and attention within a unified architecture. This design enhances the segmentation of irregular tumor thresholds and small lesions while sustaining good computational efficiency. The ablation study moreover illustrates that the outcome enhancement is obtained through the integrated contribution of all presented modules. The three-level pre-processing improves CT image quality, the A-R block enhances local trait separation, BM-BOA refines encoder traits before character fusion, and DTARM optimizes hierarchical trait illustrations. The stable enhancements obtained after the inclusion of each module contribute to the efficiency of the whole DTARNU-Net architecture.

### Critical Analysis of DTARNU-Net

As opposed to the conventional attention-based U-Net framework employing trait refinement, this approach utilizes trait fusion, followed by hierarchical attention optimization. This series strategy enhances trait illustration and maintains a fine anatomical threshold. In contrast to transformer-based segmentation models, DTARNU-Net also requires less computational power due to BM-BOA refining only the encoder trait demonstrations, replacing the implemented world-wide self-attention to all feature maps. Besides these benefits, the presented architecture has some thresholds. The inclusion of BM-BOA slightly enhances computational complexity and presents hyperparameters which want careful tuning. However, the presented model has been examined only on the LiTS threshold dataset. Overall, the results illustrate excellent segmentation efficiency, although validation on individual datasets and prospective medical studies are needed before the model can be employed for regular medical utilization.

The introduced DTARNU-Net model demonstrates the difficulties of precise liver tumor segmentation in CT images utilizing the LiTS threshold dataset. The process starts with a three-level pre-processing pipeline that involves histogram equalization, noise removal, image smoothing, and image sharpening. This pre-processing enhances CT image quality and improves tumor visibility, enabling the model to detect small or poorly formed liver tumors more precisely. The DTARNU-Net framework integrates a nested-U-Net with developed trait learning to separate rich multi-scale illustrations for precise segmentation. Further, the A-R block incorporated into the convolutional layers improves local trait extraction and enhances the diagnosis of tumors with only slight variations in pixel intensity. This supports the model in maintaining significant boundary data and precisely segmenting small or indistinct tumors that are frequently difficult to distinguish from the surrounding healthy liver tissue.

**Figure 9:**
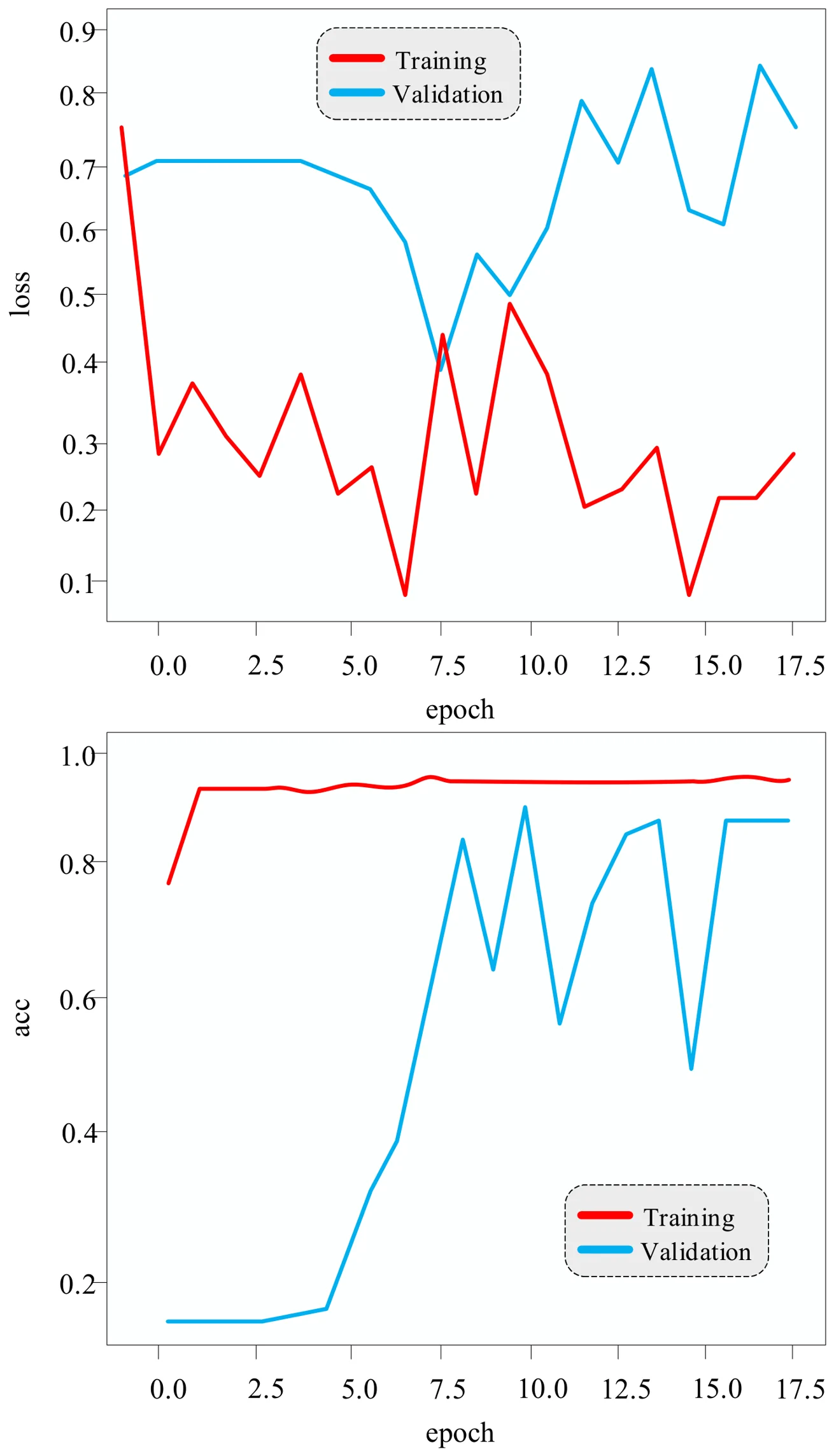
Accuracy performance analysis.

The presented DTARNU-Net moreover enhances segmentation by integrating various trait types, which include histogram, texture, and binary and RST features, to acquire a more intelligent detection of the CT images. Among the proposed elements, DTARM is an important methodological introduction, as it successfully optimizes hierarchical traits while maintaining spatial data through residual attention learning. All experimental conclusions proposed in the study were consistent when utilizing the LiTS threshold dataset, illustrating the efficiency of DTARNU-Net under a constant examination protocol. Future work will examine the presented architecture on additional public datasets, like 3d-IRCADb-01 and CHAOS, for further assessments of its normalization capability. Figure 9 shows the training and validation loss.

An extra key element is the collaboration with BM-BOA that refines encoder traits before trait fusion, enhancing trait representation and maintaining significant semantic data. This enables the model to precisely detect difficult tumor thresholds and obtain more accurate liver tumor segmentation. In contrast to the prevailing methods, BM-BOA improves character refinement without incurring excessive computational challenges. Also, the use of Brownian motion enhances the exploration ability of the optimization procedures, decreases premature convergence, and creates more robust trait illustration than conventional butterfly refinement algorithms. The presented DTARNU-Net reliably surpasses the currently prevailing liver tumor segmentation models, including ResUNet, AIM-UNet, ERes-UNet++, Rdctrans U-Net, and ARU-Net, across various examination metrics like the Dice coefficient, Accuracy, mIoU, and FWIoU. These enhancements illustrate the model’s capability to generate more precise and reliable liver tumor segmentation, specifically in difficult cases with abnormal tumor thresholds. Contrasted with currently prevailing methods, DTARNU-Net provides multiple benefits. ResUNet may lose valuable threshold information due to its heavy dependence on residual connections. AIM-UNet can err from overtraining and decreased normalization when applied to unseen datasets. ERes-UNet++ may encounter difficulties with large differences in liver shape and irregular anatomy. In contrast, DTARNU-Net integrates adaptive trait refinement with hierarchical attention optimization to maintain fine threshold data, enhance trait indications, and generate more sustaining segmentation outcomes among various liver tumor features.

Rdctrans U-Net utilizes a hybrid variable architecture that integrates convolutional and transformer-based traits. Overall, this design enhances adaptability; it may generate variable segmentation conclusions among various liver CT datasets due to the challenges in optimizing its network elements. In comparison, DTARNU-Net is particularly designed for liver tumor segmentation, in which the presented modules work together to enhance trait learning and precisely segment small and abnormal tumors. ARU-Net, which incorporates EfficientNet with an attention-based residual U-Net, obtains good segmentation effectiveness but has relatively high computational difficulty. This can present a threshold for its real-time utilization or resource-constrained medical environments. DTARNU-Net, on the other hand, integrates developed attention mechanisms with adaptive trait refinement to enhance computational efficiency, while at same time sustaining high segmentation accuracy. The limitation of this study is that the presented DTARNU-Net was examined only on the LiTS dataset. Overall, LiTS is a dataset broadly used for liver tumor segmentation; further validation on individual datasets, like 3D-IRCAB-01 and CHAOS, is necessary to better evaluate the model’s normalization capability. Upcoming work will concentrate on cross-dataset examination and domain adjustment to examine the robustness of the presented architecture under various imaging protocols and patient populations. Finally, DTARNU obtains good stability among segmentation precision, feature demonstration, and computational effectiveness. The integration of BM-BOA and DTARM facilitates effective trait refinement and hierarchical attention learning, differentiating the presented model from the currently prevailing CNN-based and transformer-based segmentation methods. As an outcome, DTARNU-Net furnishes more precise and reliable segmentation of liver tumors, specifically those with abnormality boundaries and heterogeneous intensity patterns, making it an encouraging method for future medical processes.

## 6. Conclusions

Liver tumor segmentation is necessary for the detection of liver cancer, treatment planning, and the monitoring of the progression of the disease. Moreover, currently prevailing segmentation methods frequently face difficulties with tumors that have abnormalities in their shapes, varying sizes, and intensity measurements similar to surrounding liver tissue, resulting in imprecise segmentation conclusions. This study presented DTARNU-Net, an innovative architecture for liver tumor segmentation, utilizing the LiTS threshold dataset. The presented method begins with a three-level pre-processing pipeline involving histogram equalization, noise removal, image smoothing, and sharpening to enhance CT image quality and improve tumor visualization. The network architecture integrates an A-R block to strengthen local trait separation and the Brownian Motion-based Butterfly Optimization Algorithm (BM-BOA) to refine encoder traits before character highlights. Considered together, these elements enhance trait learning, sustain tumor thresholds, and improve segmentation precision. The ablation study verifies that every presented module contributes to the integrated performance. The progressive addition of the three-level pre-processing pipeline, A-R block, BM-BOA, and DTARM sustainably enhanced the segmentation results, illustrating the efficiency of their integrated design. Experimental examination on the LiTS dataset demonstrated that DTARNU-Net obtained 96.78% Precision for liver segmentation and a 97.00% Dice score for liver tumor segmentation, exceeding multiple currently prevailing state-of-the-art methods. These outcomes illustrate that the presented architecture supports accurate, reliable, and computationally effective liver tumor segmentation, making it a valuable approach for computer-assisted detection and treatment planning. As opposed to conventional refinement methods that update only network parameters during training, BM-BOA refines hierarchical trait highlights prior to multi-scale trait fusion, enabling the decoder to use many discriminating semantic data elements. Integrated with the hierarchical attention optimization of DTARM, this framework enhances threshold localization and segmentation robustness while sustaining computational effectiveness.

Altogether, the presented model obtained excellent effectiveness; in particular, all experiments were performed on the LiTS dataset. Additional validation of DTARNU-Net with public datasets like 3D-IRCADb-01 and CHAOS remains for future work, in addition to research on cross-dataset normalization, domain adaptation, and border clinical examination. This support will also enhance the robustness and practical relevance of the presented architecture for liver tumor segmentation and other clinical image segmentation tasks.

## Figures and Tables

**Table 1:** Backbone network.

A-R	Size (Channel × Height × Width)	
	Input	Output
$D_{A}^{0,0}$	3 × 256 × 256	48 × 256 × 256
$D_{B}^{0,0}$	3 × 256 × 256	48 × 256 × 256
$D^{0,1}$	192 × 256 × 256	48 × 256 × 256
$D^{0,2}$	240 × 256 × 256	48 × 256 × 256
$D^{0,3}$	288 × 256 × 256	48 × 256 × 256
$D^{0,4}$	336 × 256 × 256	48 × 256 × 256
$D_{A}^{1,0}$	48 × 128 × 128	96 × 128 × 128
$D_{B}^{1,0}$	48 × 128 × 128	96 × 128 × 128
$D^{1,1}$	384 × 128 × 128	96 × 128 × 128
$D^{1,2}$	480 × 128 × 128	96 × 128 × 128
$D^{1,3}$	576 × 128 × 128	96 × 128 × 128
$D_{A}^{2,0}$	96 × 64 × 64	192 × 64 × 64
$D_{B}^{2,0}$	96 × 64 × 64	192 × 64 × 64
$D^{2,1}$	768 × 64 × 64	192 × 64 × 64
$D^{2,2}$	960 × 64 × 64	192 × 64 × 64
$D_{A}^{3,0}$	192 × 32 × 32	384 × 32 × 32
$D_{B}^{3,0}$	192 × 32 × 32	384 × 32 × 32
$D^{3,1}$	1536 × 32 × 32	384 × 32 × 32
$D_{B}^{4,0}$	384 × 16 × 16	768 × 16 × 16

**Table 2:** Quantitative comparison with state-of-the-art methods on the LiTS Dataset.

Method	Dice (%)	IoU (%)	Accuracy (%)	Precision (%)	Recall (%)	F1-Score (%)
ResUNet	94.21	91.68	95.14	94.06	94.38	94.22
AIM-Unet	94.86	92.54	95.61	94.73	95.02	94.87
Eres-UNet++	95.48	93.31	95.98	95.26	95.64	95.45
Rdctrans U-Net	95.87	94.02	96.21	95.78	95.93	95.85
ARU-Net	96.23	94.58	96.47	96.12	96.36	96.24
DTARNU-Net	97.00	95.82	96.78	96.43	97.15	97.00

**Table 3:** Ablation study of the proposed DTARNU-Net.

Configuration	Dice (%)	IoU (%)	Accuracy (%)
Baseline UNet++	92.81	90.46	94.32
+Three-level Preprocessing	93.88	91.54	95.13
+A-R Residual Block	94.95	92.71	95.98
+BM-BOA	95.96	94.15	96.54
+DTARM (Proposed DTARNU-Net)	97.00	95.82	96.78

## Data Availability

The data used in this study are openly available from public sources. No new data were generated.

## References

[1] Ni Y., Chen G., Feng Z., Cui H., Metaxas D.N., Zhang S., Zhu W. Domain-Adversarial Transformer Network for Multiphase Liver Tumor Segmentation. Proceedings of the 2023 45th Annual International Conference of the IEEE Engineering in Medicine & Biology Society (EMBC).

[2] Zhu L., Liu S. (2025). DGA-Net: A dual-branch group aggregation network for liver tumor segmentation in medical images. Front. Med. Technol..

[3] Wu C., Chen Q., Wang H., Guan Y., Mian Z., Huang C., Ruan C., Song Q., Jiang H., Pan J. (2024). A review of deep learning approaches for multimodal image segmentation of liver cancer. J. Appl. Clin. Med. Phys..

[4] Yashaswini G.N., Manjunath R.V., Shubha B., Prabha P., Aishwarya N., Manu H.M. (2025). Deep Learning Technique for automatic Liver and Liver Tumor Segmentation in CT Images. J. Liver Transplant..

[5] Rahman H., Aoun N.B., Bukht T.F., Ahmad S., Tadeusiewicz R., Pławiak P., Hammad M. (2025). Automatic liver tumor segmentation of CT and MRI volumes using ensemble ResUNet-InceptionV4 model. Inf. Sci..

[6] Wan Z., Zhang X., Jiang Y., Chai S., Lyu C., Hu Y. (2025). Deep learning in hepatic oncology imaging: A narrative review of computed tomography applications. Oncologie.

[7] Manghi I., Pecchi A., Esposito G., Macheda M., Zanni E., Di Benedetto F., Ferraguti F. (2025). Deep learning for automatic segmentation of hepatocellular carcinoma in contrast enhanced CT scans. Sci. Rep..

[8] Goceri E. (2025). A hybrid attention-based deep learning model for segmentation of livers and liver tumors from CT scans. Multimed. Tools Appl..

[9] Abdoli N., Sadri Y., Gilley P.W., Zhang K., Chen Y., Qiu Y. (2025). From CNNs to SAM: A Survey of Deep Learning Techniques for Liver Tumor Segmentation in CT Images. IEEE Access Pract. Innov. Open Solut..

[10] Balaguer-Montero M., Marcos Morales A., Ligero M., Zatse C., Leiva D., Atlagich L.M., Staikoglou N., Viaplana C., Monreal C., Mateo J. (2025). A CT-based deep learning-driven tool for automatic liver tumor detection and delineation in patients with cancer. Cell Rep. Med..

[11] Manjunath R., Gowda N.Y., Lakkannavar M.C. (2025). An automated deep learning model for liver tumor and skin lesion segmentation. Discov. Electron..

[12] Gao W. (2025). Liver Health: A Deep Learning-Based Auxiliary Diagnostic System for Liver Tumor Segmentation in CT Images. Acad. J. Comput. Inf. Sci..

[13] Shin H., Han K., Lee S., Park H., Kim S., Kim J., Yang X., Yang J.D., Song J., Yu H.C. (2026). Deep Learning-Based Liver Tumor Segmentation from Computed Tomography Scans with a Gradient-Enhanced Network. Diagnostics.

[14] Karabağ B., Ayturan K., Hardalaç F. (2026). Liver Tumor Segmentation with Deep Learning: A Comparative Analysis of CNN-, Transformer-, and YOLO-Based Models on the ATLAS MRI. Diagnostics.

[15] Li X., Jia W., Sharipov K., Ruslan A., Mazbutdzhon L., Shuhratjon I., Zheng Y. (2025). Precise Liver Tumor Segmentation in CT Using a Hybrid Deep Learning-Radiomics Framework. arXiv.

[16] Ghorbani N., Jamshidi B., Rostamy-Malkhalifeh M. (2025). Enhanced Liver Tumor Detection in CT Images Using 3D U-Net and Bat Algorithm for Hyperparameter Optimization. arXiv.

[17] Jha D., Tomar N.K., Biswas K., Durak G., Medetalibeyoğlu A., Antalek M., Velichko Y., Ladner D.P., Borhani A.A., Bagci U. CT Liver Segmentation Via PVT-Based Encoding and Refined Decoding. Proceedings of the 2024 IEEE International Symposium on Biomedical Imaging (ISBI).

[18] Alqahtani N., Khan A.A., Mahendran R.K., Faheem M. (2025). VSMI^2^-PANet: Versatile Scale-Malleable Image Integration and Patch Wise Attention Network With Transformer for Lung Tumour Segmentation Using Multi-Modal Imaging Techniques. CAAI Trans. Intell. Technol..

[19] Khan S., Khan A.A., Mahendran R.K., Fazil M., Rehman A.U., Jiang W., Farouk A. (2025). C2DEEP-OT: Utilizing Multi-Agent Deep Reinforcement Learning Algorithm and Optimized Attentive Transformer Network for Cervical Cancer Detection. Inf. Sci..

[20] Wang F., Hou J., Wu S. A liver tumor segmentation method based on cascaded fully convolutional neural networks and 3D conditional random fields. Proceedings of the Third International Conference on Artificial Intelligence and Electromechanical Automation.

[21] Manjunath R.V., Kwadiki K. (2022). Automatic liver and tumour segmentation from CT images using Deep learning algorithm. Results Control Optim..

[22] Rahman H., Bukht T.F.N., Imran A., Tariq J., Tu S., Alzahrani A. (2022). A deep learning approach for liver and tumor segmentation in CT images using ResUNet. Bioengineering.

[23] Özcan F., Uçan O.N., Karaçam S., Tunçman D. (2023). Fully automatic liver and tumor segmentation from CT image using an AIM-Unet. Bioengineering.

[24] Hussain M., Saher N., Qadri S. (2022). Computer vision approach for liver tumor classification using CT dataset. Appl. Artif. Intell..

[25] Li J., Liu K., Hu Y., Zhang H., Heidari A.A., Chen H., Zhang W., Algarni A.D., Elmannai H. (2023). Eres-UNet++: Liver CT image segmentation based on high-efficiency channel attention and Res-UNet++. Comput. Biol. Med..

[26] Lei T., Wang R., Zhang Y., Wan Y., Liu C., Nandi A.K. (2021). DefED-Net: Deformable encoder-decoder network for liver and liver tumor segmentation. IEEE Trans. Radiat. Plasma Med. Sci..

[27] Zhang Y., Peng C., Peng L., Huang H., Tong R., Lin L., Li J., Chen Y.W., Chen Q., Hu H. (2021). Multi-phase liver tumor segmentation with spatial aggregation and uncertain region inpainting. Medical Image Computing and Computer Assisted Intervention–MICCAI 2021: 24th International Conference, Strasbourg, France, September 27–October 1, 2021, Proceedings, Part I 24.

[28] Sabir M.W., Khan Z., Saad N.M., Khan D.M., Al-Khasawneh M.A., Perveen K., Abdul Q., Azhar Ali S.S. (2022). Segmentation of liver tumor in CT scan using ResU-Net. Appl. Sci..

[29] Han K., Liu L., Song Y., Liu Y., Qiu C., Tang Y., Teng Q., Liu Z. (2022). An effective semi-supervised approach for liver CT image segmentation. IEEE J. Biomed. Health Inform..

[30] Aghamohammadi A., Ranjbarzadeh R., Naiemi F., Mogharrebi M., Dorosti S., Bendechache M. (2021). TPCNN: Two-path convolutional neural network for tumor and liver segmentation in CT images using a novel encoding approach. Expert Syst. Appl..

[31] Li L., Ma H. (2022). Rdctrans u-net: A hybrid variable architecture for liver ct image segmentation. Sensors.

[32] Hong Y., Mao X.W., Hui Q.L., Ouyang X.P., Peng Z.Y., Kong D.X. (2021). Automatic liver and tumor segmentation based on deep learning and globally optimized refinement. Appl. Math.-A J. Chin. Univ..

[33] Wang J., Zhang X., Lv P., Wang H., Cheng Y. (2022). Automatic liver segmentation using EfficientNet and Attention-based residual U-Net in CT. J. Digit. Imaging.

[34] Ni Y., Chen G., Feng Z., Cui H., Metaxas D., Zhang S., Zhu W. (2024). DA-Tran: Multiphase liver tumor segmentation with a domain-adaptive transformer network. Pattern Recognit..

[35] Khoshkhabar M., Meshgini S., Afrouzian R., Danishvar S. (2023). Automatic Liver Tumor Segmentation from CT Images Using Graph Convolutional Network. Sensors.

[36] Bi R., Ji C., Yang Z., Qiao M., Lv P., Wang H. (2022). Residual based attention-Unet combing DAC and RMP modules for automatic liver tumor segmentation in CT. Math. Biosci. Eng..

[37] Napte K., Mahajan A., Urooj S. (2023). Automatic Liver Cancer Detection Using Deep Convolution Neural Network. IEEE Access.

[38] Liu H., Yang J., Jiang C., He S., Fu Y., Zhang S., Hu X., Fang J., Ji W. (2024). S2DA-Net: Spatial and spectral-learning double-branch aggregation network for liver tumor segmentation in CT images. Comput. Biol. Med..

[39] Nallasivan G., Ramachandran V., Alroobaea R., Almotiri J. (2023). Liver Tumors Segmentation Using 3D SegNet Deep Learning Approach. Comput. Syst. Sci. Eng..

